# Supplementary material for: A randomised controlled trial of fluoxetine versus naltrexone in compulsive sexual behaviour disorder: presentation of the study protocol
Source: BMJ Open. 2022 Jun 3;12(6):e051756. doi: 10.1136/bmjopen-2021-051756 (PMC9171192; doi:10.1136/bmjopen-2021-051756)
Supplement: Supplementary data [file bmjopen-2021-051756supp001.pdf]

INFORMED CONSENT FORM

Pharmacological treatement of Compulsive Sexual Behavior

- a comparison of the effectiveness of naltrexon versus fluoxetin: the CeSar-study

Background and aim

Here at ANOVA clinic we treat persons who experience a troublesome or dangerous sexuality. There is a lack of knowledge regarding treatment of intrusive sexual thoughts, impulses and behaviors in individuals with compulsive sexuality, sometimes called "sexual addiction". This study is evaluating a new drug for this purpose, naltrexone. Naltrexone is currently used in the treatment of alcohol dependence and has been reported as a drug that can also reduce both urges and behaviors linked to compulsive sexual behavior. To compare the new treatment, half of the participants will instead receive fluoxetine which is used for the treatment of compulsive sexuality nationally and internationally.
The overall purpose of this study is to improve the treatment options and the quality of life for the individual.

Request for participation

You have contacted or been referred to ANOVA and are therefore asked to participate. Participation is voluntary.

How is the study conducted?

If you are interested in participating in the study and provide an informed consent in the web platform, you will be asked to complete an online survey for approximately 60-90 minutes with questions on your sexuality and your general well-being. The online survey is the first step in the study and aims to provide increased knowledge about sexuality that leads to negative consequences.
After you have filled in the online survey, you will be contacted by mail or telephone and offered an appointment at the clinic ANOVA. You will meet a physician and psychologist for two interviews. They will evaluate if you meet the requirements to be enrolled in the study and you will have the opportunity to ask questions. If you want to proceed with the study, you will be requested to sign an informed consent for participation in the drug treatment. You will also be asked to participate in a computerized test of impulsivity and to submit a routine blood test to map sex hormones and liver-, thyroid- and kidney function and metabolic status (= 5 tubes, approximately 20ml) before treatment. We also ask for a urine sample to have objective measures to exclude a recreational drug use. This urine sample is discarded if it is negative or sent to a laboratory for further analysis if positive as done with routine sample in healthcare. If you are a woman of childbearing age, you will have to submit a sample (urine or blood sample, a 3.5ml tube) to rule out pregnancy and are encouraged to use a safe contraception method during the study.

We also ask if you are willing to provide research blood samples (max. 100ml) with the aim to examine neurobiological markers of compulsive sexuality. These samples include e.g. DNA extraction, see further under the heading Biobank below.

Estimated time required for the visits is about 3 hours.

If you are offered treatment within the framework of the study, you will receive treatment for 8 weeks with either naltrexone or fluoxetine. The selection is made through randomization (i.e. assignment by chance). You will know which of the preparations you have been randomized to, but will not have the opportunity to choose drug.

You will be contacted by phone after 3-5 days and after 4 weeks to inquire about mood and possibly adjust the dose. At 4 weeks, you will be asked to provide new blood samples (3 tubes, approx. 12 ml).
You will be asked to report any side effects and if you notice any changes since the start of medication in the web-based platform. In addition, you will be asked to fill questionnaires every week that assess you mood and your sexual problems. Filling in questionnaires will take about 10-20 minutes per week.
You will be contacted and offered a psychologist or physicians' appointment if we become concerned about your mental or physical state.

Visit 2 at ANOVA: After finishing treatment, you will have a follow-up visit with a physician. You will also be asked to provide urine / blood to see the concentration of the drug in the body, routine blood tests (4 tubes, approx. 16 ml) and research blood samples (2 tubes, approx. 8 ml) to examine whether the treatment has i.e. affected hormone levels.

Visit 3 at ANOVA: Six weeks after the end of treatment, you have a final appointment with a physician who follows up on your mood and your sexuality.

Visits 2 and 3 take about 30-60 minutes each.

If you need care after the trail, you will be guided to the type of care that best suits you. Regardless of whether you are offered treatment or not, your answers in the online survey will be an important part of the study.

Biobank

Routine blood samples will be handled and destroyed according to the hospital's guidelines as in normal sampling in healthcare. The samples that are intended to be stored (5 tubes, max 30 ml) for e.g. DNA analysis and measurement of the hormone oxytocin will be stored in a biobank. The responsible biobank is Stockholms Medicinska Biobank (reg. No. 914) in accordance with the Act on Biobanks in Health Care (2002: 297), which regulates the manner

in which samples may be saved and used, and it also regulates the quality and safety of biobanks.
The samples will be stored coded, which means that the samples cannot be directly traced to you as a person. The samples and the associated identification list (code key) will be kept separate from each other, and protected from access by unauthorized persons.

Coded samples may be sent for analysis both within Sweden and the EU / USA.

Future projects: The blood samples will be stored pending analysis for up to 10 years, after which they will be destroyed. The samples may also be relevant for not yet unplanned research projects if you approve storage for this. A new ethical application will be made in such case, and you may be contacted again with a request for your consent. A separate request regarding future projects is provided in the enclosed consent form.

If you regret that you gave permission for your sample to be stored, you have the right to have the samples destroyed or deidentified by contacting test leader Josephine Savard, please find contact information below.

Are there any risks associated with the study?

Answering questions on private topics such as sexuality and mental health might feel uncomfortable, but these questions need to be asked so we can help you.
As for the medicine, you can experience side effects that are usually transient such as nausea, headache, vomiting and weakness. Serious side effects are uncommon with both drugs. For safety reasons, you will have to provide blood samples before and after the treatment to map, among other things, liver function. Should your samples be abnormal, we will process it according to medical practice. If you are a woman of reproductive age, you need to use safe contraception throughout the study period and submit a negative pregnancy test to be included in the study.

While blood sampling, you may experience temporary discomfort, dizziness and you may get a bruise.

During the course of the study, you could use drugs temporary such as painkillers (e.g. paracetamol) or short-term anti-anxiety medication (e.g. Atarax). Other treatment should be avoided and may lead to discontinuation from the study. This specially applies to drugs with addictive features. Ongoing drug treatment is usually suitable to continue with, but dose changes should be avoided. If in doubt, discuss with your treating physician at ANOVA.

Warning # 1! Should you develop symptoms such as jaundice / hepatitis (inflammation of the liver), you must immediately contact an emergency department or call 112. Symptoms of jaundice are feelings of illness, yellow staining of the skin and whites of the eyes, abdominal pain, light stools and yellow urine.

Warning # 2! Naltrexone counteracts the effect of opioids found in e.g. painkillers (Citodon, OxyContin /OxyNorm, Dolcontin, morphine, etc.) and in cough medicines (Cocillana-Etyfin). There are also opioids in loperamide (Dimor, Loperamide, Imodium) used to treat diarrhea. Should an emergency situation arise where you need treatment with opioids, you should immediately stop taking the study drug and inform the responsible physician about the simultaneous participation in this study.

Warning # 3! Should you during the course of treatment deteriorate mentally with e.g. suicidal ideation, you must contact the study physiatrist or psychiatric emergency service at your place of residence. You will receive contact information at the start of study.

We recommend that you always carry the plastic card you are assigned at start of the study. The card has contact information to the study supervisor so that the treatment unit can obtain information about the study

Are there any benefits to participating in the study?

There is a possibility that you will feel relieved to have shared your thoughts and feelings with a professional and that the drug takes the edge off the desire to commit sexual acts. It can also be an advantage to have contacted our clinic to enroll as a "regular patient" after the study period is over.

Data management and confidentiality

Information about you will be registered within the framework of the study:
- Self-assessments are handled in a web-based platform that is encrypted and protected with double authentication requirements on a secure server at Karolinska Institutet.
- Other data such as interview forms and biobank samples will be handled pseudonymised, i.e. linked to you through a study code. The code key will be kept at ANOVA and protected from access by unauthorized persons.

Your answers and results will be stored in locked folders, secure digital files, encrypted databases and servers in accordance with the General Data Protection Regulation (GDPR 2016/619). Your information will not be used commercially and will be stored for a maximum of 10 years after the end of the study. All information will be handled with total confidentiality. It will not be possible to link any information to you as an individual when the results are reported.

As in ANOVA's regular care, computer-based patient records will be generated. These will not be visible outside ANOVA's record domain.

In order to verify that the collected data is handled correct, and that the procedures are performed in accordance with applicable laws and regulations, the implementation of the research study will be reviewed by an independent trial investigator. Investigators who review

the research study may be given access to decoded data and patient records together with your responsible physician and under confidentiality.

Personal data is protected and processed in accordance with the General Data Protection Regulation (GDPR 2016/679) and the Public Access to Information and Secrecy Act (2009: 400). No unauthorized person will be allowed to access the information. According to the General Data Protection Regulation, you have the right to obtain the information that is handled of you in the study free of charge and, if necessary, have any errors corrected.

If you want to take part of the information, you can contact test leader Dr Josephine Savard for more information, contact information can be found on the last page under the section Responsible. Responsible for the management of personal data is Karolinska University Hospital's Data Protection Office. The Data Protection Officer can be reached at: Karolinska University Hospital, 171 76 Solna, tel. 08-517 700 00 (switchboard operator), e-mail: dataskyddsombud.karolinska@regionstockholm.se. If you are dissatisfied with how your personal data is processed, you have the right to submit a complaint to the Swedish Authority for Privacy Protection, which is the supervisory authority.

How do I get information about the results of the study?

The results will be published in scientific journals. Only statistical variables will be presented, and it will not be possible to identify individual participants. If you want to take part of the scientific reports, you are welcome to contact ANOVA where this study will be carried out. Information on clinical trials can be found at: www.clinicaltrials.gov

Insurance and compensation

The regular patient insurance applies. The drugs and the visits will be free of charge.

Participation is voluntary

Participation in the study is voluntary and you can withdraw your participation at any time without stating why and without it affecting your future care at ANOVA. If you want to discontinue your participation, contact the study supervisor or the test leader (contact information below). After you withdraw your participation, no new information about you will be saved. Already collected data will be preserved.
Samples: If you regret that you gave permission for us to store your samples, you have the right to have the samples discarded (this means that the samples will be destroyed or deidentified).

Responsible for the trial

Responsible for the implementation of the study is ANOVA, Karolinska University Hospital. The principal researcher is Professor Jussi Jokinen (jussi.jokinen@ki.se)

For questions, please contact test leader Josephine Savard, research nurse Susanne Jarlvik Alm or research assistant Pia Jaensson

Dr. Josephine Savard (Testleader)
ANOVA, Karolinska Universitetssjukhuset, 171 76 Stockholm.
E-post: josephine.savard@regionstockholm.se, Tel +46 (0)72-5823241

Susanne Jarlvik Alm
ANOVA, Karolinska Universitetssjukhuset, 171 76 Stockholm.
E-post: Susanne.jarlvik-alm@regionstockholm.se, Tel +46 (0) 8-51772935

Pia Jaensson
ANOVA, Karolinska Universitetssjukhuset, 171 76 Stockholm.
E-post: pia.jaensson@regionstockholm.se, Tel +46 (0) 8-51773832

Consent form: Drug treatment for compulsive sexuality, CeSar study.

• I have read the written information regarding the research study and I have had the opportunity to ask questions and have them answered.
• I provide my consent to participate in the study and know that my participation is completely voluntary. I am aware that blood and urine samples need to be provided based on clinical indication. I regulate how the samples are saved (see below).
• I am aware that I can withdraw my consent and terminate my participation at any time and without explanation, as well as having collected samples discarded. The samples will then be destroyed or deidentified.
• I allow my personal information to be registered according to the information I have received and that collected data about me is stored and handled electronically by study supervisors. I allow unidentified data to be analyzed in future, unplanned studies after approval by the Ethics Review Authority.
• I allow the study supervisor or a study monitor to receive patient record information that is relevant to the current research study and that the Swedish Medical Products Agency and the corresponding supervisory authority within and outside the EU receive patient record information during any supervision.

Signature Name in block letters Date

---------------------------- --------------------------------- --------------------

Consent, research on blood samples within the framework of the study: I give my consent that the samples I submit will be saved in the biobank and that the samples are used for research in accordance with what is described in the patient consent information.

Yes □ No □

Consent, preservation of samples for future research studies: Your samples may be valuable for unplanned research projects (as described in this information) if you approve storage for this. In these cases, a new ethical review will take place and you may be contacted again with a request. Do you consent to that samples that remain after the completion of the Cesar study are stored for future research and may be analyzed within Sweden, the EU and the USA?

Yes □ No □
Signature Name in block letters Date

------------------------------ --------------------------------- --------------------

To be completed by the investigator: I confirm that I have provided both oral and written information about the described trial and that a copy of the patient information sheet has been handed out to the patient.

Signature Name in block letters Date

------------------------------ ---------------------------------- --------------------
