# Supplementary material for: A randomised controlled trial of fluoxetine versus naltrexone in compulsive sexual behaviour disorder: presentation of the study protocol
Source: BMJ Open. 2022 Jun 3;12(6):e051756. doi: 10.1136/bmjopen-2021-051756 (PMC9171192; doi:10.1136/bmjopen-2021-051756)
Supplement: Supplementary data [file bmjopen-2021-051756supp002.pdf]

Supplementary Table
Outcome Measures with Psychometric Properties

Name	Psychometric properties
The Mini International Neuropsychiatric Interview (MINI)	Validated<br>(Sheehan et al., 1998).
Hypersexual Disorder: Current Assessment Scale (HD:CAS)	Not validated<br>Cronbach's α = 0.76<br>(Savard., 2021)
The Hypersexual Behavior Inventory (HBI)	Validated<br>test-retest reliability *r* = 0.91; Cronbach's *α* = 0.96<br>(Reid et al., 2011)
Self-assessment of Sexual Interest (SSI)	Not validated<br>(Långström, unpublished)
The Hypersexual Disorder Screening Inventory (HDSI)	Validated in Swedish<br>inter-rater reliability *r* = 0.51, Cronbach's α = 0.80-0.81<br>(Öberg et al., 2017)
International Index of Erectile Function (IIEF)	Validated<br>test–retest reliability *r* = 0.64–0.84; Cronbach's α = 0.73–0.99) across studies.<br>(Rosen et al., 1997; 2002)
Sexual Compulsivity Scale (SCS)	Cronbach's α = 0.89-0.92<br>(Kalichman & Rompa, 1995)
The Alcohol Use Disorders Identification Test (AUDIT)	Validated in Swedish<br>test-retest reliability *r* = 0.97; Cronbach's α 0.82<br>(Bergman & Kallmen, 2002)
The Drug Use Disorders Identification Test (DUDIT)	Validated in Swedish<br>Cronbach's α = 0.80; sensitivity (ranging from 0.85- 1.00) and specificity (ranging from 0.75 to 0.92)<br>(Berman et al., 2005; Hildebrand 2015)
The Gambling Disorder Identification Test (G-DIT)	Test-retest reliability<br>intraclass correlation coefficient = 0.93<br>Cronbach's α = 0.94<br>(Molander et. al., 2021)
The Childhood Trauma Questionnaire – Short Form (CTQ-SF)	Validated in Swedish<br>The inter-correlations between CTQ total scale and subscales vary between *r* = 0.15-0.89; Cronbach's α = 0.92 on the total score, subscales α = 0.65-0.86<br>(Bernstein & Fink, 1998; Gerdner & Allgulander 2009)

Karolinska Interpersonal Violence Scale (KIVS)	Validated in Swedish<br>inter-rater reliability for the subscales *r* = 0.91-0.95<br>(Jokinen et al., 2010)
Adult ADHD Self-Report Scale (ASRS)	Validated<br>test-retest reliability *r* = 0.58-0.77<br>(Kessler et al., 2007)
The Barratt Impulsiveness Scale (BIS)	Validated<br>test-retest reliability Spearman's rho = 0.83<br>Cronbach's *α* = 0.83<br>(Stanford et al., 2009)
Montgomery Åsberg Depression Rating Scale – Self-rating (MADRS-S)	Validated in Swedish<br>inter-rater reliability *r* = 0.87; Cronbach's α = 0.84<br>(Svanborg & Asberg 2001; Fantino, B., & Moore, N. 2009)
Hospital Anxiety and Depression Scale (HAD)	Validated in Swedish<br>test-retest reliability *r* = 0.72; Cronbachs α = 0.89-0.93<br>(Lisspers, J., et al. 1997)
Columbia Suicide Severity Rating Scale (C-SSRS)	Validated in Swedish<br>C-SSRS total score, AUC = 0.65 and a cut-off of 28.5 gave a sensitivity of 69% and a specificity of 54% in predicting a non-fatal or fatal suicide attempt<br>Cronbach's α = 0.73-0.95<br>(Posner et al., 2011; Lindh et al., 2018)
UKU side effect rating scale (UKU)	Validated<br>inter-rater reliability *r* = 0.07-0.80<br>(Lingjaerde et al., 1987; Lindström 2001)

Notes: Bergman, H., & Kallmen H. (2002). Alcohol use among Swedes and a psychometric evaluation of the alcohol use disorders identification test. Alcohol and Alcoholism, 37(3), 245–251. https://doi.org/10.1093/alcalc/37.3.245

Berman, A. H., Bergman, H., Palmstierna, T., & Schlyter, F. (2005). Evaluation of the Drug Use Disorders Identification Test (DUDIT) in Criminal Justice and Detoxification Settings and in a Swedish Population Sample. European Addiction Research, 11(1), 22-31. https://doi.org/10.1159/000081413

Bernstein DP, Fink L. Childhood Trauma Questionnaire. A retrospective self-report. Manual. The Psychological Corporation, Harcourt Brace & Company, San Antonio, TX 1998

Fantino, B., & Moore, N. (2009). The self-reported Montgomery-Asberg Depression Rating Scale is a useful evaluative tool in Major Depressive Disorder. BMC psychiatry, 9, 26. https://doi.org/10.1186/1471-244X-9-26

Gerdner, A., & Allgulander, C. (2009). Psychometric properties of the Swedish version of the Childhood Trauma Questionnaire-Short Form (CTQ-SF). Nordic journal of psychiatry, 63(2), 160–170. https://doi.org/10.1080/08039480802514366

Hildebrand M. (2015). The Psychometric Properties of the Drug Use Disorders Identification Test (DUDIT): A Review of Recent Research. Journal of substance abuse treatment, 53, 52–59. https://doi.org/10.1016/j.jsat.2015.01.008

Jokinen, J., Forslund, K., Ahnemark, E., Gustavsson, J. P., Nordström, P., & Asberg, M. (2010). Karolinska Interpersonal Violence Scale predicts suicide in suicide attempters. The Journal of clinical psychiatry, 71(8), 1025–1032. https://doi.org/10.4088/JCP.09m05944blu

Kalichman, S. C., & Rompa, D. (1995). Sexual sensation seeking and Sexual Compulsivity Scales: reliability, validity, and predicting HIV risk behavior. Journal of personality assessment, 65(3), 586–601. https://doi.org/10.1207/s15327752jpa6503_16

Kessler, R. C., Adler, L. A., Gruber, M. J., Sarawate, C. A., Spencer, T., & Van Brunt, D. L. (2007). Validity of the World Health Organization Adult ADHD Self-Report Scale (ASRS) Screener in a representative sample of health plan members. International journal of methods in psychiatric research, 16(2), 52–65. https://doi.org/10.1002/mpr.208

Lindh, Å.U., Waern, M., Beckman, K. et al. Short term risk of non-fatal and fatal suicidal behaviours: the predictive validity of the Columbia-Suicide Severity Rating Scale in a Swedish adult psychiatric population with a recent episode of self-harm. BMC Psychiatry 18, 319 (2018). https://doi.org/10.1186/s12888-018-1883-8

Lindström, E., Lewander, T., Malm, U., Malt, U. F., Lublin, H., & Ahlfors, U. G. (2001). Patient-rated versus clinician-rated side effects of drug treatment in schizophrenia. Clinical validation of a self-rating version of the UKU Side Effect Rating Scale (UKU-SERS-Pat). Nordic journal of psychiatry, 55 Suppl 44, 5–69. https://doi.org/10.1080/080394801317084428

Lingjaerde, O., Ahlfors, U. G., Bech, P., Dencker, S. J., & Elgen, K. (1987). The UKU side effect rating scale. A new comprehensive rating scale for psychotropic drugs and a cross-sectional study of side effects in neuroleptic-treated patients. Acta psychiatrica Scandinavica. Supplementum, 334, 1–100. https://doi.org/10.1111/j.1600-0447.1987.tb10566.x

Lisspers, J., Nygren, A., & Söderman, E. (1997). Hospital Anxiety and Depression Scale (HAD): some psychometric data for a Swedish sample. Acta psychiatrica Scandinavica, 96(4), 281–286. https://doi.org/10.1111/j.1600-0447.1997.tb10164.x

Långström N. Self-assessment of Sexual Interest (SSI). Unpublished questionnaire. 2010.

Molander, O., Wennberg, P., & Berman, A. H. (2021). The Gambling Disorders Identification Test (GDIT): Psychometric Evaluation of a New Comprehensive Measure for Gambling Disorder and Problem Gambling. Assessment, 10731911211046045. Advance online publication. https://doi.org/10.1177/10731911211046045

Posner, K., Brown, G. K., Stanley, B., Brent, D. A., Yershova, K. V., Oquendo, M. A., Currier, G. W., Melvin, G. A., Greenhill, L., Shen, S., & Mann, J. J. (2011). The Columbia-Suicide Severity Rating Scale: initial validity and internal consistency findings from three multisite studies with adolescents and adults. The American journal of psychiatry, 168(12), 1266–1277. https://doi.org/10.1176/appi.ajp.2011.10111704

Reid, R. C., Garos, S., & Carpenter, B. N. (2011). Reliability, Validity, and Psychometric Development of the Hypersexual Behavior Inventory in an Outpatient Sample of Men. Sexual Addiction & Compulsivity, 18(1), 30-51. https://doi.org/10.1080/10720162.2011.555709

Rosen, R. C., Riley, A., Wagner, G., Osterloh, I. H., Kirkpatrick, J., & Mishra, A. (1997). The international index of erectile function (IIEF): a multidimensional scale for assessment of erectile dysfunction. Urology, 49(6), 822–830. https://doi.org/10.1016/s0090-4295(97)00238-0

Rosen, R. C., Cappelleri, J. C., & Gendrano, N., 3rd (2002). The International Index of Erectile Function (IIEF): a state-of-the-science review. International journal of impotence research, 14(4), 226–244. https://doi.org/10.1038/sj.ijir.3900857

Savard, J. (2021). Compulsive sexual behavior disorder: clinical characteristics and treatment with Naltrexone (PhD dissertation, Umeå universitetet). Retrieved from http://urn.kb.se/resolve?urn=urn:nbn:se:umu:diva-186811

Sheehan, D. V., Lecrubier, Y., Sheehan, K. H., Amorim, P., Janavs, J., Weiller, E., Hergueta, T., Baker, R., & Dunbar, G. C. (1998). The Mini-International Neuropsychiatric Interview (M.I.N.I.): the development and validation of a structured diagnostic psychiatric interview for DSM-IV and ICD-10. Journal of Clinical Psychiatry, 59 Suppl 20, 22-33;quiz 34-57

Stanford, M. S., Mathias, C. W., Dougherty, D. M., Lake, S. L., Anderson, N. E., & Patton, J. H. (2009). Fifty years of the Barratt Impulsiveness Scale: An update and review. Personality and Individual Differences, 47(5), 385–395. https://doi.org/10.1016/j.paid.2009.04.008

Svanborg P, Asberg M. A comparison between the Beck Depression Inventory (BDI) and the self-rating version of the Montgomery Asberg Depression Rating Scale (MADRS). J Affect Disord. 2001;64(2-3):203-16.

Öberg, K. G., Hallberg, J., Kaldo, V., Dhejne, C., & Arver, S. (2017). Hypersexual Disorder According to the Hypersexual Disorder Screening Inventory in Help-Seeking Swedish Men and Women With Self-Identified Hypersexual Behavior. Sexual medicine, 5(4), e229–e236. https://doi.org/10.1016/j.esxm.2017.08.001
