# Supplementary material for: A randomised controlled trial of fluoxetine versus naltrexone in compulsive sexual behaviour disorder: presentation of the study protocol
Source: BMJ Open. 2022 Jun 3;12(6):e051756. doi: 10.1136/bmjopen-2021-051756 (PMC9171192; doi:10.1136/bmjopen-2021-051756)
Supplement: Author's manuscript [file bmjopen-2021-051756.draft_revisions.pdf]

BMJ Open

BMJ Open is committed to open peer review. As part of this commitment we make the peer review history of every article we publish publicly available.

When an article is published we post the peer reviewers' comments and the authors' responses online. We also post the versions of the paper that were used during peer review. These are the versions that the peer review comments apply to.

The versions of the paper that follow are the versions that were submitted during the peer review process. They are not the versions of record or the final published versions. They should not be cited or distributed as the published version of this manuscript.

BMJ Open is an open access journal and the full, final, typeset and author-corrected version of record of the manuscript is available on our site with no access controls, subscription charges or pay-per-view fees (http://bmjopen.bmj.com).

If you have any questions on BMJ Open's open peer review process please email info.bmjopen@bmj.com

BMJ Open

A Randomized Controlled Trial of Fluoxetine Versus Naltrexone in Compulsive Sexual Behavior Disorder: Presentation of the Study Protocol

Journal:	*BMJ Open*
Manuscript ID	bmjopen-2021-051756
Article Type:	Protocol
Date Submitted by the Author:	27-Mar-2021
Complete List of Authors:	Savard, Josephine; Umea University, Department of Clinical Sciences/psychiatry; Karolinska University Hospital, ANOVA<br>Görts Öberg, Katarina; Karolinska University Hospital, ANOVA; Karolinska Institute, Department of Medicine<br>Dhejne, Cecilia; Karolinska University Hospital, ANOVA; Karolinska Institute, Department of Medicine<br>Jokinen, Jussi; Umea University, Department of Clinical Sciences/psychiatry; Karolinska Institute, Centre for Psychiatry Research, Department of Clinical Neuroscience
Keywords:	Impulse control disorders < PSYCHIATRY, Sexual and gender disorders < PSYCHIATRY, Adult psychiatry < PSYCHIATRY

SCHOLARONE™
Manuscripts

Title Page

A Randomized Controlled Trial of Fluoxetine Versus Naltrexone in Compulsive Sexual Behavior Disorder: Presentation of the Study Protocol

Date for submission

03/27/2021

Authors

Family name(s) are typed in **bold**

Josephine **Savard**, MD[a, b] josephine.savard@umu.se

Katarina **Görts Öberg**, PhD[b,c] katarina.gorts-oberg@sll.se

Cecilia **Dhejne**, MD, PhD[b,c] cecila.dhejne@sll.se

Jussi **Jokinen**, MD, PhD[a, d] jussi.jokinen@ki.se, jussi.jokinen@umu.se

[a] Department of Clinical Sciences/Psychiatry, Umeå University, Umeå, Sweden.

[b] Anova, Karolinska University Hospital, Stockholm, Sweden.

[c] Department of Medicine, Karolinska Institutet, Stockholm, Sweden.

[d] Centre for Psychiatry Research, Department of Clinical Neuroscience, Karolinska Institutet, Stockholm, Sweden.

Corresponding author: Josephine Savard, ANOVA, Karolinska University Hospital, 171 76 Stockholm, Sweden.

Telephone: (+46)8-517 732 00. Fax number: (+46)8-517 718 14. E-mail: josephine.savard@umu.se

Word count: 2625

For peer review only

ABSTRACT

Background: Compulsive Sexual Behavior Disorder is a new disorder in the ICD-11, and is associated with negative consequences in different areas of life. Evidence for pharmacological treatment of compulsive sexual behavior disorder is weak and treatment options are limited. This proposed study will be the largest and the first randomized controlled trial comparing the efficacy and tolerability of two active drugs in Compulsive Sexual Behavior Disorder.

Methods and analysis: Eighty adult participants with Compulsive Sexual Behavior Disorder according to ICD-11 will be randomized to receive either Naltrexone 25-50 mg or Fluoxetine 20-40 mg for eight weeks, followed by six weeks without treatment. The study will be conducted in a sub-specialized outpatient sexual medicine unit at Karolinska University Hospital, Stockholm, Sweden. The study is financed by grants and entirely independent of the manufacturers.

Exclusion criteria include severe psychiatric or psychical illness, changes to concurrent medication, and non-compatible factors contraindicating the use of either drug. The primary outcome measure is the Hypersexual Disorder: Current Assessment Scale (HD: CAS), and tolerability will be assessed by the UKU side effect rating scale, drug accountability, adherence to treatment, and drop-out rate. Participants will complete questionnaires at regular intervals, with the main endpoint for efficacy after 8 weeks (end of treatment) and after 14 weeks (follow-up). Blood chemistry will be repeatedly collected as a safety precaution and for research purposes. The results will be analyzed using an appropriate analysis of variance model or a mixed model, depending on the distribution of HD: CAS and the extent of missing data.

Ethics and dissemination: The Swedish Ethical Review Authority and the Swedish Medical Products Agency have approved the study on 27 May 2020 and 4 June 2020, respectively (Ref. no. 2020-02069 and Ref. no. 5.1-2020-48282). Findings will be published in peer-reviewed journals and presented at relevant conferences.

Registration details: Clinicaltrialsregister.eu, EudraCT: 2019-004255-36 (Cesar study).

For peer review only

Strengths and limitations of this study

This randomized controlled trial will compare the efficacy of two pharmacological approaches of Compulsive Sexual Behavior Disorder.

The results will contribute to evidence-based pharmacological treatment of Compulsive Sexual Behavior Disorder.

The study protocol duration of only 14 weeks is a limitation, nevertheless deemed sufficient to provide important clinical knowledge.

Although we aim to have a representative sample, a certain impact on the external validity is to be expected.

INTRODUCTION

Compulsive Sexual Behavior Disorder (CSBD) is a newly-defined diagnosis in the impulse control disorder section of the 11th edition of the International Classification of Diseases (ICD-11).[1] It entails a persistent pattern of failure to control intense repetitive sexual impulses or urges, resulting in sexual behaviors with negative consequences affecting different areas of life. Adverse consequences associated with CSBD include distress, unwanted pregnancies, sexually transmitted diseases, relationship problems, financial expenses, occupational impairment, and risk of crime. Considering these potential severe consequences and the suggested prevalence of 3-6% of the population,[2] there is an undisputable, urgent need for effective treatments.

Existing drug studies of CSBD have mainly been case reports and small open-label studies on Selective Serotonin Reuptake Inhibitors (SSRIs),[3-4] anti-androgens,[4] or the opioid antagonist naltrexone.[5-8] Only one randomized controlled trial has examined the effect of an SSRI (citalopram 20-60 mg/day for 12 weeks) in 28 gay and bisexual men with non-paraphilic compulsive sexual behavior.[9] There was no significant difference in the main outcome measure Y-BOCS-CSB score between those assigned citalopram and those assigned placebo.

For peer review only

CSBD and Paraphilic Disorders are regarded as separate conditions, however, a high level of sexual pre-occupation is common in both.[10-13] To aid physicians in clinical practice, the World Federation of Societies of Biological Psychiatry (WFSBP) has provided treatment guidelines for paraphilic disorders.[14-15] Psychotherapy is proposed as a first step for low risk individuals. If results are unsatisfactory, the next step includes SSRIs.

The subsequent levels in the guidelines are for individuals with moderate-high risk for sexual violence and include testosterone-lowering agents such as cyproterone acetate (CPA) and gonadotropin-releasing hormone agonists. These agents decrease the frequency and intensity of sexual desire and arousal, however use is associated with high rates of adverse effects. Hormonal treatment is potentially unsuitable for help-seeking individuals with conventional CSBD. In order to investigate appropriate pharmacological alternatives, the proposed 14-week randomized controlled trial will compare the efficacy of two active drugs in 80 adults with CSBD. Additional aims are to examine tolerability and what clinical characteristics and biomarkers could be predictors of response.

Based on the WFSBP treatment guidelines and available research,[9] the SSRI *fluoxetine* will be defined as standard treatment. Alternative treatment will consist of *naltrexone*, an opioid antagonist which prevents reinforcing effects in the mesolimbic reward center and is used in the treatment of alcohol use disorder.[16] Naltrexone was chosen due to similarities between CSBD and other urge-driven disorders, and promising results in CSBD case reports, and in our pilot study of 20 men conducted during 2018-2019.[17] In the latter, all participants completed the eight week study protocol, and no serious adverse events occurred. Although adverse reactions were common, all were considered mild to moderate, and most were transient during the first days-week. Hence, we were able to determine that naltrexone was a well-tolerated, feasible treatment option, and gave indications of symptom relief. However, as the study format prohibited conclusion of efficacy, this study aims to further investigate naltrexone in the treatment of CSBD.

Primary Objective

To compare the effect of naltrexone versus fluoxetine in Compulsive Sexual Behavior Disorder.

For peer review only

Secondary Objectives

To investigate if clinical, psychosocial or biological factors can predict treatment response.

To compare if there are any differences in drop-out rate and adherence between the two treatment groups.

To assess side effects and investigate any connection between drop-out rate, reports of side effects and/or efficacy.

To compare if there is a difference in the participants' wish to resume treatment.

To investigate biological markers and clinical parameters in participants with Compulsive Sexual Behavior Disorder.

To investigate correlation between Compulsive Sexual Behavior Disorder and impulsiveness, experience of violence and suicidality.

METHIODS AND ANALYSIS

Study setting and study design

The study will be conducted at ANOVA, a subspecialized sexual medicine outpatient clinic at Karolinska University Hospital in Stockholm, Sweden. Eligible participants will be randomized in blocks with a 1:1 allocation to receive either fluoxetine or naltrexone in a parallel group design for eight weeks followed by six week follow-up phase. Blinding will not be applicable due to the different appearance of the drugs and dose augmentation in different time intervals due to different pharmacological properties.

Protocol version

Version identifier: 2.0. Number of protocol amendments: 1; 25 June 2020; main changes included adding blood samples and assessments for safety reasons as requested by the Swedish Medical Products Agency.

For peer review only

Eligibility Criteria

Please see Table 1 for inclusion and exclusion criteria.

Table 1. Inclusion and exclusion criteria

**Inclusion criteria**
Meet criteria for Compulsive Sexual Behavior Disorder according to ICD-11 and fulfill criteria for Hypersexual Disorder as originally proposed for inclusion in DSM-5.
Between 18-65 years.
Understand oral and written Swedish and have internet access.
Willing to participate in all study visits including providing blood and urine samples.
Signed informed consent form.
For fertile women: the use of a safe method of contraception during the entire study protocol.
**Exclusion criteria**
Signs of hepatitis, elevated liver enzymes (> 3 times over reference), or a history of liver failure.
eGFR < 60 ml/min, signs or history of acute kidney failure.
fp-glucose ≥ 7.0 mmol/l.
Known heart disease such as angina pectoris, previous heart failure, or heart attack.
Other serious physical illness including diabetes mellitus, epilepsy, or known ocular hypertension.
Treatment in the past month (≥ 1 dose) with opioids or benzodiazepines.
Treatment in the past month with oral anticoagulants such as warfarin. Intermittent treatment (max. 15 doses per week) with NSAID (e.g. ibuprofen) is tolerated.
Treatment with tamoxifen.
Self-reported use of recreational drugs in the past month or positive drug verification analysis.
Alcohol dependence or risk consumption (> 14 units of alcohol per week for men, > 9 for women) in the past month.
Severe psychiatric disorder requiring immediate treatment such as current psychotic disorder or severe depression.
Bipolar disorder or history of hypomania.
Ongoing treatment with naltrexone or SSRI, or previous hypersensitivity reaction to either.
Change of concurrent medication or dosage in the past three months regarding antidepressants, ADHD medication, mood stabilizers, antipsychotics, cortisone, testosterone, or dopamine precursors. Smaller adjustments may in some cases be acceptable (assessed by study psychiatrist).

For peer review only

Ongoing pharmacological treatment with contraindicated substances (e.g. tamoxifen, metoprolol).
Pregnancy and/or breastfeeding.
Mental condition that could negatively influence either the participant's health or the scientific aspects of the study. High risk for committing sexual offense is included.
Ongoing psychotherapeutic treatment.
Participation in other studies outside ANOVA.
Abbreviations: ADHD = Attention-Deficit/Hyperactivity Disorder, NSAID = Nonsteroidal anti-inflammatory agents, DSM-5 = Diagnostic and Statistical Manual of Mental Disorders, Fifth Edition.

Recruitment, randomization and allocation

An initial screening regarding compulsive sexual behavior and inclusion and exclusion criteria (Table 1) will be conducted when potential candidates call the national helpline PrevenTell for individuals with self-identified compulsive sexual behavior and/or paraphilia. They will receive information about the study and be invited to log into a secure web-based platform to leave a preliminary informed consent and fill in questionnaires.

Participants will thereafter attend a baseline visit at the clinic. After obtaining written informed consent, urine samples will be collected and screened for recreational drug use (e.g. amphetamine, benzodiazepines, cannabis, cocaine, and opioid) and blood samples will be collected (e.g. monitoring of testosterone, LH, glucose, electrolytes, and liver enzymes) (Table 2).

Table 2. Samples analyzed during the study

**Screening**		
S-FSH	P-ASAT	P-Sodium
S-LH	P-Calcium	
S-SHBG	fP-Glucose	Full blood cell count
S-Testosterone	P-GT	B-HbA1c

For peer review only

S-TSH	P-HDL Cholesterol	
S-T4	P-Potassium	U-screening for substance abuse [a]
P-Albumin	P-Cholesterol	U-Pregnancy test [b]
P-ALAT	P-Creatinine	
P-ALP	fP-LDL Cholesterol	Research samples [c]
**At 4 weeks**		
P-ALAT	P-GT	Full blood cell count
P-ALP	P-Potassium	
P-ASAT	P-Creatinine	U-Pregnancy test [b]
P-Glucose	P-Sodium	
**At 8 weeks**		
P-ALAT	P-Creatinine	U-Pregnancy test [b]
P-ALP	P-Sodium	P-Fluoxetine or U-Naltrexone [d]
P-ASAT	Full blood cell count	
P-GT	B-HbA1c	Research samples [c]
P-Potassium		

Notes: [a] Amphetamine, benzodiazepines, buprenorphine, cannabis, cocaine, fentanyl, methadone, oxycodone, tramadol, and other opioids.
[b] Assigned female at birth only
[c] e.g. DNA extraction for genome-wide methylation analysis (EPIC) and second generation DNA sequencing, oxytocin pre- and post-treatment.
[d] Depending on randomization

A psychiatrist will obtain a medical and psychiatric history, perform a physical examination including blood pressure and heart and pulmonary auscultation, as well as perform interviews with the Mini International Neuropsychiatric Interview (MINI)[18] and Columbia Suicide Severity Rating Scale (C-SSRS).[19] The study psychologist will focus on sexual behaviors by conducting a structured interview addressing the ICD-11 criteria for CSBD and the Diagnostic and Statistical Manual of Mental

For peer review only

Disorders, Fifth Edition (DSM-5) criteria for Hypersexual Disorder as originally proposed for inclusion[20] and paraphilia(s). The separate psychiatrist and psychologist interviews aim to determine eligibility criteria in an unbiased manner. If unmatched opinions, the participant will not be included.

The research nurse will open envelopes for randomization and supply enrolled participants with either naltrexone or fluoxetine accordingly. Participants will start treatment if results on blood chemistry are adequate.

The study drugs will be provided by the Karolinska University Hospital's pharmacy.

Naltrexone (AOP Orphan Pharmaceuticals): Initial dose 25 mg per day, and if tolerated will be augmented to 50 mg per day after 3-5 days.

Fluoxetine (Orion Pharma): Initial dose 20 mg per day, and if unsatisfactory symptom reduction and tolerated will be augmented to 40 mg per day after four weeks.

In Sweden, The Dental and Pharmaceutical Benefits Agency, a central government agency determines what pharmaceutical product shall be subsidized by the state. The agency also recommend manufacturers for specific drugs based on e.g. prize and availability. The manufacturers for naltrexone and fluoxetine in this study were chosen as they were the agency's recommended product when the application was prepared for submission

Intervention

Figure 1 illustrates study procedures. Participants will assess their symptoms weekly by filling in questionnaires online e.g. if they have noticed any change in frequency or intensity of sexual urges or behaviors, how they perceive the treatment and if they experience adverse reactions. Every second week, participants will complete the main outcome measure HD: CAS. After four weeks, new blood samples will be collected and participants will have a telephone consultation with the study psychiatrist in order to assess tolerability and psychiatric well-being. Fertile women will be required to conduct a pregnancy test before starting the study and use contraception during the study period.
At end of treatment, participants will have a consultation with the study psychiatrist to assess current symptoms of CSBD, psychiatric distress, and tolerability. Blood samples will be collected for chemical analysis as well as monitoring of the metabolites of naltrexone respective fluoxetine.
Six weeks after the end of treatment, the participants will fill in questionnaires and have a final consultation with the study psychiatrist.

For peer review only

Outcome measures

The main outcome is symptom relief as assessed with The Hypersexual Disorder: Current Assessment Scale (HD: CAS),[21] which measures symptom severity during the previous two weeks according to the suggested conceptualization of Hypersexual Disorder to the DSM-5.[20] Corresponding scales for the ICD-11 diagnosis have not yet been developed. We chose a scale that is sensitive to changes and participants will be asked to fill it in at baseline, every second week during the treatment phase, and at end of study. However, as the scale is not validated, two additional measures will be used: the Hypersexual Behavior Inventory (HBI)[22] and the Sexual Compulsivity Scale (SCS).[23] Using unpublished data from our research center, the correlations (Pearson's) between HD: CAS and HBI were positive at baseline and post-treatment.

We will also analyze factors (clinical, psychosocial, or biological) that may be predictors of response, and whether there is any difference in participants' readiness to resume pharmacological treatment at end of study.

Furthermore, we will also evaluate tolerability using the UKU side effect rating scale,[24] drug accountability, adherence to treatment, and drop-out rate.

Finally, to aid in understanding CSBD, we will assess for impulsivity, suicidality, and childhood adversities, and collect biological markers (e.g. DNA and hormones) to evaluate their potential association with CSBD. A summary of assessments and biological markers is presented in Table 2 and 3. Furthermore, an extension of the study is planned including neuroimaging data collection.

Table 3. Study assessments

**Objective**	**Assessments**	**Measure type**
Assess changes in compulsive sexual behaviors from screening to week 8 and follow up	Hypersexual Disorder: Current Assessment Scale (HD: CAS).	Outcome
Assess compulsive sexual behaviors	The Hypersexual Disorder Screening Inventory (HDSI)	Eligibility
Assess changes in compulsive sexual behaviors from screening to week 8 and follow up	The Hypersexual Behavior Inventory (HBI)	Outcome
Assess changes in compulsive sexual behaviors from screening to week 8 and follow up	Sexual Compulsivity Scale (SCS)	Outcome

For peer review only

Assess sexual interests	Self-rating sexual interests (SSI)	Eligibility
Assess sexual dysfunction	International Index of Erectile Function (IIEF)[a]	Eligibility
Assess psychiatric co-morbidity	Mini International Neuropsychiatric Interview (MINI)	Eligibility
Assess changes in depression severity from screening to week 8 and follow up	Montgomery Åsberg Depression Rating Scale – Self-rating (MADRS-S)	Outcome Safety
Assess change of symptoms of depression and anxiety from screening to week 8 and follow up	Hospital Anxiety and Depression Scale (HAD)	Outcome
Assess changes in alcohol use from screening to week 8 and follow up	Alcohol Use Disorders Identification Test (AUDIT)	Outcome
Assess use of drugs	Drug Use Disorders Identification Test (DUDIT)	Eligibility
Assess pathological gambling	Gambling Disorder Identification Test (G-DIT)	Eligibility
Assess impulsivity	Barratt Impulsiveness Scale (BIS-11)	Eligibility
Assess symptoms of Attention-Deficit/ Hyperactivity Disorder	Adult ADHD Self-Report Scale (ASRS)	Eligibility
Assess history of violence	Karolinska Interpersonal Violence Scale (KIVS)	Eligibility
Assess history of childhood trauma	The Childhood Trauma Questionnaire – Short Form (CTQ-SF)	Eligibility
Assess suicide ideation and suicidal behavior throughout the study	Columbia Suicide Severity Rating Scale (C-SSRS)	Safety
Assess adverse reactions in week 8	UKU side effect rating scale (UKU)	Tolerability
Question of wanting to resume pharmacological treatment, week 14	Yes/No/Don't know option	Outcome
Notes: [a] assigned men at birth only.		

Adherence and Concomitant Care

Participants can leave the study at any time without giving a reason, however any material already obtained will be analyzed. Participants who miss ≥ 3 doses in a row will be considered as having ceased treatment. Other reasons for discontinuation of treatment include severe adverse reactions,

For peer review only

severe psychiatric condition where the safety of the participant or others cannot be guaranteed, initiation of treatment with non-compatible drugs or use of recreational drugs, pregnancy, or start of psychotherapy.

Sample Size and Statistical Methods

Since there is no gold standard for the measurement of pharmacological treatment outcomes in CSBD, the sample size calculation is based on HD: CAS from a study of Cognitive Behavioral Therapy from our clinic[25] (using expanded data available post-publication, n = 76). The difference in HD: CAS pre-post treatment was 4.065 with a standard deviation of 4.74. A statistical power of 80 %, an error probability of 0.05, and a difference between the groups of 3.3 (meaning an effect size of 0.7) would render a sample size of 34 participants in each group. To adjust for potential dropouts we aim to include 80 participants. The results will be analyzed using an appropriate analysis of variance model or a mixed model, depending on the distribution of HD: CAS and the extent of missing data. Participants who discontinue treatment will be included in an intention-to-treat analysis. Per-protocol analysis will also be used. To increase the study's credibility, the extent and nature of missing data will be reported for each treatment group, as well as any predictors of missing data (e.g. young age).

Monitoring

As required, an independent trial investigator from the Karolinska Trial Alliance will regularly control and quality assure the study procedures. There will be no interim analysis.

Patient and public involvement

Patients and the public will not be involved in the design of the study. Advertisement in media will be made.

All subjects will be informed about the study both orally and in writing, including information on the potential benefits and risks of the therapies, and informed consent will be collected. The visits at the clinic and the drugs will be free of charge. Participants will be informed about the study results on a group level.

Ethics and dissemination

The study procedures will be carried out in accordance with the Declaration of Helsinki and European Good Clinical Practice Guidelines. The Swedish Ethical Review Authority and the Swedish Medical Products Agency have approved the study (Ref. no. 2020-02069 and Ref. no. 5.1-2020-48282). The registration of the trial (Eudra-CT no. 2019-004255-36) is accessible at https://www.clinicaltrialsregister.eu. Study enrollment started in October 2020.

Eligible participants will be aged ≥ 18 years and understand both oral and written Swedish. Paraphilic interests are not an exclusion criterion, whereas severe risk for sexual violence (as reported in the

For peer review only

interviews and questionnaires, e.g. participants who report that they actively interact with children for sexual purposes) is.

The study psychiatrists and psychologists are experienced in evaluating and treating patients with CSBD and Paraphilic Disorders, and the investigators will together decide whether the individual may be a potential study candidate.

The risks for the participants including safety aspects of the drugs have been carefully weighed against the potential benefits of conducting the study, and we believe the latter outweighs the former. The participants' health and safety will be assessed and prioritized, which may entail discontinuation of treatment.

Research data from the web-based platform will be stored securely in servers with restricted access. Archived paper material will stored in a locked cabinet, in accordance with current legislation and regulations. Research samples and data will be destroyed 10 years after declaring end of trial to the Swedish Medical Products Agency.

The results of the study will be presented at scientific conferences and in peer-review articles.

DISCUSSION

As presented in the introduction, there is currently weak evidence for the pharmacological treatment of CSBD (for review, see Grubbs et al.[26]) and hence a clear need for randomized controlled trials. However, some aspects of this study design and concept need to be discussed:

Feasibility of enrollment

Participants might be unwilling to participate in a drug trial, hence it may be difficult to complete recruitment within the designated time period of three years, leading to a sample too small for rending power in the analyses. In addition, the Covid-19 pandemic might complicate participants' travel to our clinic or even our capacity to conduct the study due to restrictions in the provision of healthcare (e.g. limited to emergency care only). An overrepresentation of participants living in the Stockholm area is to be expected.

Eligibility

We try to minimize the risk for recruiting a homogeneous group of persons with CSBD by inviting individuals with a broad range of severity of symptoms, and the use of randomization with allocation concealment to minimize the risk for selection bias between the groups. Nevertheless, as with most studies, there is a risk for an overrepresentation of those with a better prognosis.

The exclusion criteria are more stringent than the inclusion criteria, partly due to the safety profiles of the drugs – a situation that regardless of study protocol would influence their use in a regular clinical

For peer review only

setting. However, we also exclude participants with ongoing recreational drug use to minimize the risk of confounders. We also exclude participants currently being treated with interacting antidepressants and those with a severe psychiatric disorder requiring immediate treatment, as these patients are too sick to follow our study protocol. Most patients seeking treatment at ANOVA for CSBD do not fall into these categories (particularly not the latter), but this exclusion criterion will undoubtedly lead to the inclusion of healthier individuals in the study. Although we aim to have a representative sample, a certain impact on the external validity is to be expected.

Outcome ascertainment

As noted, our main outcome variable HD: CAS is not validated. However, it has a predefined timespan of symptom relief and has been used in previous studies at our research center. When comparing results of HD: CAS with the validated HBI, the latter reaches a floor-effect post treatment, whereas HD: CAS seems to better distinguish effects. Our overall judgement is thus that the advantages of the HD: CAS outweigh the disadvantages.

Another limitation is that this study will not be blinded, and we will not be able to control for the anticipation effect in either group. Nevertheless, group comparison values using regression models with incorporated baseline will still be of major value, and results will nonetheless be of importance to guide clinical praxis and for future meta-analyses. Finally, the study protocol duration of only 14 weeks is a major limitation, but the length chosen for feasibility reasons as well as being deemed sufficient to provide important clinical knowledge.

Acknowledgements
The authors wish to thank Tania McConaghy for language revision.

Contributors

JS, KGÖ, CD and JJ conceived and designed the study. JS and JJ contributed to the statistical plan. JJ is grant holder. JS drafted the manuscript, and all author contributed substantially to refinement of the manuscript. All authors approved the final version.

Funding
This work was supported by the Swedish Research Council grant number 2020-01183 and regional agreements between ANOVA, Region Stockholm, and Region Västerbotten (ALF). The pharmaceutical companies are not providing funding or being involved in any other way.

Competing interests

Jussi Jokinen has participated in Advisory Board of Janssen concerning esketamine for MDD with current suicidal ideation. The other authors report no conflicts of interest.

For peer review only

Figure 1. Flowchart.

Note: * If unsatisfactory symptom reduction and tolerated.

REFERENCES

1. World Health Organization. The 11th revision of the International Classification of Diseases (ICD-11). Available from: https://icd.who.int/browse11/l-m/en#/http%3a%2f%2fid.who.int%2ficd%2fentity%2f274880002 accessed February 9, 2020.
2. Kuzma JM, Black DW. Epidemiology, prevalence, and natural history of compulsive sexual behavior. *Psychiatr Clin North Am* 2008;31(4):603-11. doi: 10.1016/j.psc.2008.06.005
3. Gola M, Potenza MN. Paroxetine Treatment of Problematic Pornography Use: A Case Series. *Journal of Behavioral Addictions* 2016;5(3):529-32. doi: 10.1556/2006.5.2016.046
4. Winder B, Lievesley R, Elliott H, et al. Evaluation of the use of pharmacological treatment with prisoners experiencing high levels of hypersexual disorder. *J Forens Psychiatry Psychol* 2018;29(1):53-71. doi: 10.1080/14789949.2017.1337801
5. Raymond NC, Grant JE, Kim SW, et al. Treatment of compulsive sexual behaviour with naltrexone and serotonin reuptake inhibitors: two case studies. *Int Clin Psychopharmacol* 2002;17(4):201-5. doi: 10.1097/00004850-200207000-00008
6. Ryback RS. Naltrexone in the treatment of adolescent sexual offenders. *J Clin Psychiatry* 2004;65(7):982-86. doi: 10.4088/JCP.v65n0715
7. Raymond NC, Grant JE, Coleman E. Augmentation with naltrexone to treat compulsive sexual behavior: A case series. *Ann Clin Psychiatry* 2010;22(1):56-62.
8. Bostwick JM, Bucci JA. Internet sex addiction treated with naltrexone. *Mayo Clin Proc* 2008;83(2):226-30. doi: 10.4065/83.2.226
9. Wainberg ML, Muench F, Morgenstern J, et al. A double-blind study of citalopram versus placebo in the treatment of compulsive sexual behaviors in gay and bisexual men. *J Clin Psychiatry* 2006;67(12):1968-73. doi: 10.4088/JCP.v67n1218
10. Kafka MP, Hennen J. The paraphilia-related disorders: an empirical investigation of nonparaphilic hypersexuality disorders in outpatient males. *J Sex Marital Ther* 1999;25(4):305-19. doi: 10.1080/00926239908404008
11. Sutton KS, Stratton N, Pytyck J, et al. Patient Characteristics by Type of Hypersexuality Referral: A Quantitative Chart Review of 115 Consecutive Male Cases. *J Sex Marital Ther* 2015;41(6):563-80. doi: 10.1080/0092623x.2014.935539
12. Kafka MP, Hennen J. Hypersexual desire in males: are males with paraphilias different from males with paraphilia-related disorders? *Sex Abuse* 2003;15(4):307-21. doi: 10.1177/107906320301500407
13. Engel J, Veit M, Sinke C, et al. Same Same but Different: A Clinical Characterization of Men with Hypersexual Disorder in the Sex@Brain Study. *J Clin Med* 2019;8(2) doi: 10.3390/jcm8020157
14. Thibaut F, De La Barra F, Gordon H, et al. The World Federation of Societies of Biological Psychiatry (WFSBP) guidelines for the biological treatment of paraphilias. *World J Biol Psychiatry* 2010;11(4):604-55. doi: 10.3109/15622971003671628
15. Thibaut F, Cosyns P, Fedoroff JP, et al. The World Federation of Societies of Biological Psychiatry (WFSBP) 2020 guidelines for the pharmacological treatment of paraphilic disorders. *The World Journal of Biological Psychiatry* 2020;21(6):412-90. doi: 10.1080/15622975.2020.1744723
16. Franck J, Jayaram-Lindstrom N. Pharmacotherapy for alcohol dependence: status of current treatments. *Curr Opin Neurobiol* 2013;23(4):692-9. doi: 10.1016/j.conb.2013.05.005
17. Savard J, Öberg KG, Chatzittofis A, et al. Naltrexone in Compulsive Sexual Behavior Disorder: A Feasibility Study of Twenty Men. *J Sex Med* 2020 doi: 10.1016/j.jsxm.2020.04.318
18. Sheehan DV, Lecrubier Y, Sheehan KH, et al. The Mini-International Neuropsychiatric Interview (M.I.N.I.): the development and validation of a structured diagnostic psychiatric interview for DSM-IV and ICD-10. *J Clin Psychiatry* 1998;59 Suppl 20:22-33;quiz 34-57.

For peer review only

19. Posner K, Brown GK, Stanley B, et al. The Columbia–Suicide Severity Rating Scale: Initial Validity and Internal Consistency Findings From Three Multisite Studies With Adolescents and Adults. *Am J Psychiatry* 2011;168(12):1266-77. doi: 10.1176/appi.ajp.2011.10111704
20. Kafka MP. Hypersexual disorder: a proposed diagnosis for DSM-V. *Arch Sex Behav* 2010;39(2):377-400. doi: 10.1007/s10508-009-9574-7 [published Online First: 2009/11/26]
21. American Psychiatric Association. Hypersexual Disorder: current assessment scale. American Psychatric Association's DSM-V workgroup on Sexual and Gender Identity Disorders 2010. Available from: http://www.dsm5.org/ProposedRevisions/Pages/proposedrevision.aspx?rid=415 accessed March 2010.
22. Reid RC, Garos S, Carpenter BN. Reliability, Validity, and Psychometric Development of the Hypersexual Behavior Inventory in an Outpatient Sample of Men. *Sexual Addiction & Compulsivity* 2011;18(1):30-51. doi: 10.1080/10720162.2011.555709
23. Kalichman SC, Rompa D. Sexual sensation seeking and Sexual Compulsivity Scales: reliability, validity, and predicting HIV risk behavior. *J Pers Assess* 1995;65(3):586-601. doi: 10.1207/s15327752jpa6503_16
24. Lindstrom E, Lewander T, Malm U, et al. Patient-rated versus clinician-rated side effects of drug treatment in schizophrenia. Clinical validation of a self-rating version of the UKU Side Effect Rating Scale (UKU-SERS-Pat). *Nord J Psychiatry* 2001;55 Suppl 44:5-69. doi: 10.1080/080394801317084428
25. Hallberg J, Kaldo V, Arver S, et al. Internet-Administered Cognitive Behavioral Therapy for Hypersexual Disorder, With or Without Paraphilia(s) or Paraphilic Disorder(s) in Men: A Pilot Study. *J Sex Med* 2020;17(10):2039-54. doi: 10.1016/j.jsxm.2020.07.018
26. Grubbs JB, Hoagland KC, Lee BN, et al. Sexual addiction 25 years on: A systematic and methodological review of empirical literature and an agenda for future research. *Clin Psychol Rev* 2020;82:101925. doi: https://doi.org/10.1016/j.cpr.2020.101925

For peer review only

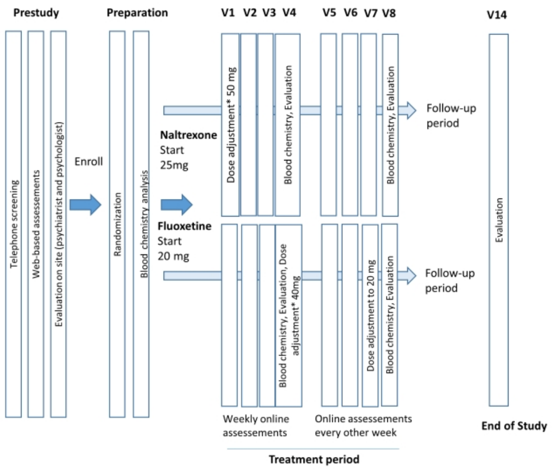

Flowchart.

338x190mm (300 x 300 DPI)

BMJ Open

A Randomized Controlled Trial of Fluoxetine Versus Naltrexone in Compulsive Sexual Behavior Disorder: Presentation of the Study Protocol

Journal:	*BMJ Open*
Manuscript ID	bmjopen-2021-051756.R1
Article Type:	Protocol
Date Submitted by the Author:	02-Feb-2022
Complete List of Authors:	Savard, Josephine; Umea University, Department of Clinical Sciences/psychiatry; Karolinska University Hospital, ANOVA<br>Görts Öberg, Katarina; Karolinska University Hospital, ANOVA; Karolinska Institute, Department of Medicine<br>Dhejne, Cecilia; Karolinska University Hospital, ANOVA; Karolinska Institute, Department of Medicine<br>Jokinen, Jussi; Umea University, Department of Clinical Sciences/psychiatry; Karolinska Institute, Centre for Psychiatry Research, Department of Clinical Neuroscience
**Primary Subject Heading**:	Mental health
Secondary Subject Heading:	Sexual health
Keywords:	Impulse control disorders < PSYCHIATRY, Sexual and gender disorders < PSYCHIATRY, Adult psychiatry < PSYCHIATRY

SCHOLARONE™
Manuscripts

Title Page

A Randomized Controlled Trial of Fluoxetine Versus Naltrexone in Compulsive Sexual Behavior Disorder: Presentation of the Study Protocol

Date for submission

03/27/2021

Authors

Family name(s) are typed in **bold**

Josephine **Savard**, MD, PhD[a,b] josephine.savard@umu.se

Katarina **Görts Öberg**, PhD[b,c] katarina.gorts-oberg@sll.se

Cecilia **Dhejne**, MD, PhD[b,c] cecila.dhejne@sll.se

Jussi **Jokinen**, MD, PhD[a, d] jussi.jokinen@ki.se, jussi.jokinen@umu.se

[a] Department of Clinical Sciences/Psychiatry, Umeå University, Umeå, Sweden.

[b] Anova, Karolinska University Hospital, Stockholm, Sweden.

[c] Department of Medicine, Karolinska Institutet, Stockholm, Sweden.

[d] Centre for Psychiatry Research, Department of Clinical Neuroscience, Karolinska Institutet, Stockholm, Sweden.

Corresponding author: Josephine Savard, ANOVA, Karolinska University Hospital, 171 76 Stockholm, Sweden.

Telephone: (+46)8-517 732 00. Fax number: (+46)8-517 718 14. E-mail: josephine.savard@umu.se

Word count: 2922

For peer review only

ABSTRACT

Background: Compulsive Sexual Behavior Disorder is a new disorder in the ICD-11, and is associated with negative consequences in different areas of life. Evidence for pharmacological treatment of compulsive sexual behavior disorder is weak and treatment options are limited. This proposed study will be the largest and the first randomized controlled trial comparing the efficacy and tolerability of two active drugs in Compulsive Sexual Behavior Disorder.

Methods and analysis: Eighty adult participants with Compulsive Sexual Behavior Disorder according to ICD-11 will be randomized to receive either Naltrexone 25-50 mg or Fluoxetine 20-40 mg for eight weeks, followed by six weeks without treatment. The study will be conducted in a sub-specialized outpatient sexual medicine unit at Karolinska University Hospital, Stockholm, Sweden. The study is financed by grants and entirely independent of the manufacturers.

Exclusion criteria include severe psychiatric or psychical illness, changes to concurrent medication, and non-compatible factors contraindicating the use of either drug. The primary outcome measure is the Hypersexual Disorder: Current Assessment Scale (HD: CAS), and tolerability will be assessed by the UKU side effect rating scale, drug accountability, adherence to treatment, and drop-out rate. Participants will complete questionnaires at regular intervals, with the main endpoint for efficacy after 8 weeks (end of treatment) and after 14 weeks (follow-up). Blood chemistry will be repeatedly collected as a safety precaution and for research purposes. The results will be analyzed using an appropriate analysis of variance model or a mixed model, depending on the distribution of HD: CAS and the extent of missing data.

Ethics and dissemination: The Swedish Ethical Review Authority and the Swedish Medical Products Agency have approved the study on 27 May 2020 and 4 June 2020, respectively (Ref. no. 2020-02069 and Ref. no. 5.1-2020-48282). Findings will be published in peer-reviewed journals and presented at relevant conferences.

Registration details: Clinicaltrialsregister.eu, EudraCT: 2019-004255-36 (Cesar study).

For peer review only

Strengths and limitations of this study

This is the first randomized controlled trial comparing the efficacy of two pharmacological treatments of Compulsive Sexual Behavior Disorder- naltrexone and fluoxetine.

The study enables assessment of clinical, psychosocial and biological predictors of treatment response.

The lack of placebo-control is a limitation.

The study protocol duration of 14 weeks may be seen as limitation of the study design.

INTRODUCTION

Compulsive Sexual Behavior Disorder (CSBD) is a newly-defined diagnosis in the impulse control disorder section of the 11th edition of the International Classification of Diseases (ICD-11).[1] It entails a persistent pattern of failure to control intense repetitive sexual impulses or urges, resulting in sexual behaviors with negative consequences affecting different areas of life. Adverse consequences associated with CSBD include distress, unwanted pregnancies, sexually transmitted diseases, relationship problems, financial expenses, occupational impairment, and risk of crime. Considering these potential severe consequences and the suggested prevalence of 3-6% of the population,[2] there is an undisputable, urgent need for effective treatments.

Existing drug studies of CSBD have mainly been case reports and small open-label studies on Selective Serotonin Reuptake Inhibitors (SSRIs),[3-4] anti-androgens,[4] or the opioid antagonist naltrexone.[5-8] Only one randomized controlled trial has examined the effect of an SSRI (citalopram 20-60 mg/day for 12 weeks) in 28 gay and bisexual men with non-paraphilic compulsive sexual behavior.[9] There was no significant difference in the main outcome measure Y-BOCS-CSB score between those assigned citalopram and those assigned placebo.

CSBD and Paraphilic Disorders are regarded as separate conditions, however, a high level of sexual pre-occupation is common in both.[10-13] To aid physicians in clinical practice, the World Federation of Societies of Biological Psychiatry (WFSBP) has provided treatment guidelines for paraphilic

For peer review only - http://bmjopen.bmj.com/site/about/guidelines.xhtml

disorders.[14-15] Psychotherapy is proposed as a first step for low risk individuals. If results are unsatisfactory, the next step includes SSRIs.

The subsequent levels in the guidelines are for individuals with moderate-high risk for sexual violence and include testosterone-lowering agents such as cyproterone acetate (CPA) and gonadotropin-releasing hormone agonists. These agents decrease the frequency and intensity of sexual desire and arousal, however use is associated with high rates of adverse effects. Hormonal treatment is potentially unsuitable for help-seeking individuals with conventional CSBD. In order to investigate appropriate pharmacological alternatives, the proposed 14-week randomized controlled trial will compare the efficacy of two active drugs in 80 adults with CSBD. Additional aims are to examine tolerability and what clinical characteristics and biomarkers could be predictors of response.

Based on the WFSBP treatment guidelines and available research,[9] the SSRI *fluoxetine* will be defined as standard treatment. Alternative treatment will consist of *naltrexone*, an opioid antagonist which prevents reinforcing effects in the mesolimbic reward center and is used in the treatment of alcohol use disorder.[16] Naltrexone was chosen due to similarities between CSBD and other urge-driven disorders, and promising results in CSBD case reports, and in our pilot study of 20 men conducted during 2018-2019.[17] In the latter, all participants completed the eight week study protocol, and no serious adverse events occurred. Although adverse reactions were common, all were considered mild to moderate, and most were transient during the first days-week. Hence, we were able to determine that naltrexone was a well-tolerated, feasible treatment option, and gave indications of symptom relief. However, as the study format prohibited conclusion of efficacy, this study aims to further investigate naltrexone in the treatment of CSBD.

Primary Objective

To compare the effect of naltrexone versus fluoxetine in Compulsive Sexual Behavior Disorder.

Secondary Objectives

To investigate if clinical, psychosocial or biological factors can predict treatment response.

For peer review only - http://bmjopen.bmj.com/site/about/guidelines.xhtml

To compare if there are any differences in drop-out rate and adherence between the two treatment groups.

To assess side effects and investigate any connection between drop-out rate, reports of side effects and/or efficacy.

To compare if there is a difference in the participants' wish to resume treatment.

To investigate biological markers and clinical parameters in participants with Compulsive Sexual Behavior Disorder.

To investigate correlation between Compulsive Sexual Behavior Disorder and impulsiveness, experience of violence and suicidality.

For peer review only

METHIODS AND ANALYSIS

Study setting and study design

The study will be conducted at ANOVA, a subspecialized sexual medicine outpatient clinic at Karolinska University Hospital in Stockholm, Sweden. Eligible participants will be randomized in blocks with a 1:1 allocation to receive either fluoxetine or naltrexone in a superiority parallel group design for eight weeks followed by six week follow-up phase. Blinding will not be applicable due to the different appearance of the drugs and dose augmentation in different time intervals due to different pharmacological properties.

Protocol version

Version identifier: 2.0. Number of protocol amendments: 1; 25 June 2020; main changes included adding blood samples and assessments for safety reasons as requested by the Swedish Medical Products Agency.

Eligibility Criteria

Please see Table 1 for inclusion and exclusion criteria.

Table 1. Inclusion and exclusion criteria

For peer review only - http://bmjopen.bmj.com/site/about/guidelines.xhtml

**Inclusion criteria**
Meet criteria for Compulsive Sexual Behavior Disorder according to ICD-11 and fulfill criteria for Hypersexual Disorder as originally proposed for inclusion in DSM-5.
Between 18-65 years.
Understand oral and written Swedish and have internet access.
Willing to participate in all study visits including providing blood and urine samples.
Signed informed consent form.
For fertile women: the use of a safe method of contraception during the entire study protocol.
**Exclusion criteria**
Signs of hepatitis, elevated liver enzymes (> 3 times over reference), or a history of liver failure.
eGFR < 60 ml/min, signs or history of acute kidney failure.
fp-glucose ≥ 7.0 mmol/l.
Known heart disease such as angina pectoris, previous heart failure, or heart attack.
Other serious physical illness including diabetes mellitus, epilepsy, or known ocular hypertension.
Treatment in the past month (≥ 1 dose) with opioids or benzodiazepines.
Treatment in the past month with oral anticoagulants such as warfarin. Intermittent treatment (max. 15 doses per week) with NSAID (e.g. ibuprofen) is tolerated.
Treatment with tamoxifen.
Self-reported use of recreational drugs in the past month or positive drug verification analysis.
Alcohol dependence or risk consumption (> 14 units of alcohol per week for men, > 9 for women) in the past month.
Severe psychiatric disorder requiring immediate treatment such as current psychotic disorder or severe depression.
Bipolar disorder or history of hypomania.
Ongoing treatment with naltrexone or SSRI, or previous hypersensitivity reaction to either.
Change of concurrent medication or dosage in the past three months regarding antidepressants, ADHD medication, mood stabilizers, antipsychotics, cortisone, testosterone, or dopamine precursors. Smaller adjustments may in some cases be acceptable (assessed by study psychiatrist).
Ongoing pharmacological treatment with contraindicated substances (e.g. tamoxifen, metoprolol).
Pregnancy and/or breastfeeding.
Mental condition that could negatively influence either the participant’s health or the scientific aspects of the study. High risk for committing sexual offense is included.

For peer review only

Ongoing psychotherapeutic treatment.
Participation in other studies outside ANOVA.
Abbreviations: ADHD = Attention-Deficit/Hyperactivity Disorder, NSAID = Nonsteroidal anti-inflammatory agents, DSM-5 = Diagnostic and Statistical Manual of Mental Disorders, Fifth Edition.

Recruitment, randomization and allocation

An initial screening regarding compulsive sexual behavior and inclusion and exclusion criteria (Table 1) will be conducted when potential candidates call the national helpline PrevenTell for individuals with self-identified compulsive sexual behavior and/or paraphilia. Persons likely to meet criteria for CSBD will receive information about the study and be invited to log into a secure web-based platform to leave a preliminary informed consent and fill in questionnaires.

Participants will thereafter attend a baseline visit at the clinic. After obtaining written informed consent (provided as supplement material), urine samples will be collected and screened for recreational drug use (e.g. amphetamine, benzodiazepines, cannabis, cocaine, and opioid) and blood samples will be collected (e.g. monitoring of testosterone, LH, glucose, electrolytes, and liver enzymes) (Table 2). Specific research samples will also be collected (e.g. DNA extraction for genome-wide methylation analysis (EPIC) and second generation DNA sequencing).

Table 2. Samples analyzed during the study

**Screening**		
S-FSH	P-ASAT	P-Sodium
S-LH	P-Calcium	
S-SHBG	fP-Glucose	Full blood cell count
S-Testosterone	P-GT	B-HbA1c

For peer review only - http://bmjopen.bmj.com/site/about/guidelines.xhtml

S-TSH	P-HDL Cholesterol	
S-T4	P-Potassium	U-screening for substance abuse [a]
P-Albumin	P-Cholesterol	U-Pregnancy test [b]
P-ALAT	P-Creatinine	
P-ALP	fP-LDL Cholesterol	Research samples [c]
**At 4 weeks**		
P-ALAT	P-GT	Full blood cell count
P-ALP	P-Potassium	
P-ASAT	P-Creatinine	U-Pregnancy test [b]
P-Glucose	P-Sodium	
**At 8 weeks**		
P-ALAT	P-Creatinine	U-Pregnancy test [b]
P-ALP	P-Sodium	P-Fluoxetine or U-Naltrexone [d]
P-ASAT	Full blood cell count	
P-GT	B-HbA1c	Research samples [c]
P-Potassium		

Notes: [a] Amphetamine, benzodiazepines, buprenorphine, cannabis, cocaine, fentanyl, methadone, oxycodone, tramadol, and other opioids.
[b] Assigned female at birth only
[c] e.g. DNA extraction for genome-wide methylation analysis (EPIC) and second generation DNA sequencing, oxytocin pre- and post-treatment.
[d] Depending on randomization

A psychiatrist will obtain a medical and psychiatric history, perform a physical examination including blood pressure and heart and pulmonary auscultation, as well as perform interviews with the Mini International Neuropsychiatric Interview (MINI)[18] and Columbia Suicide Severity Rating Scale (C-SSRS).[19] The study psychologist will focus on sexual behaviors by conducting a structured interview addressing the ICD-11 criteria for CSBD and the Diagnostic and Statistical Manual of Mental

For peer review only - http://bmjopen.bmj.com/site/about/guidelines.xhtml

Disorders, Fifth Edition (DSM-5) criteria for Hypersexual Disorder as originally proposed for inclusion[20] and paraphilia(s). The separate psychiatrist and psychologist interviews aim to determine eligibility criteria in an unbiased manner. If unmatched opinions, the participant will not be included.

The research nurse will open envelopes for randomization and supply enrolled participants with either naltrexone or fluoxetine accordingly. The allocation sequence is created by an independent investigator at the Karolinska Trial Alliance, a research center that supports clinical trials. Participants will start treatment if results on blood chemistry are adequate.

The study drugs will be provided by the Karolinska University Hospital's pharmacy.

Naltrexone (AOP Orphan Pharmaceuticals): Initial dose 25 mg per day, and if tolerated will be augmented to 50 mg per day after 3-5 days.

Fluoxetine (Orion Pharma): Initial dose 20 mg per day, and if unsatisfactory symptom reduction and tolerated will be augmented to 40 mg per day after four weeks.

In Sweden, The Dental and Pharmaceutical Benefits Agency, a central government agency determines what pharmaceutical product shall be subsidized by the state. The agency also recommend manufacturers for specific drugs based on e.g. prize and availability. The manufacturers for naltrexone and fluoxetine in this study were chosen as they were the agency's recommended product when the application was prepared for submission

Intervention

Figure 1 illustrates study procedures. Participants will assess their symptoms weekly by filling in questionnaires online e.g. if they have noticed any change in frequency or intensity of sexual urges or behaviors, how they perceive the treatment and if they experience adverse reactions. Every second week, participants will complete the main outcome measure HD: CAS. After four weeks, new blood samples will be collected and participants will have a telephone consultation with the study psychiatrist in order to assess tolerability and psychiatric well-being. Fertile women will be required to conduct a pregnancy test before starting the study and use contraception during the study period.
At end of treatment, participants will have a consultation with the study psychiatrist to assess current symptoms of CSBD, psychiatric distress, and tolerability. Blood samples will be collected for chemical analysis as well as monitoring drug tablet return and the metabolites of naltrexone respective fluoxetine.

For peer review only - http://bmjopen.bmj.com/site/about/guidelines.xhtml

Six weeks after the end of treatment, the participants will fill in questionnaires and have a final consultation with the study psychiatrist.

Outcome measures

The main outcome is symptom relief as assessed with The Hypersexual Disorder: Current Assessment Scale (HD: CAS),[21] which measures symptom severity during the previous two weeks according to the suggested conceptualization of Hypersexual Disorder to the DSM-5.[20] Corresponding scales for the ICD-11 diagnosis have not yet been developed. We chose a scale that is sensitive to changes and participants will be asked to fill it in at baseline, every second week during the treatment phase, and at end of study. However, as the scale is not validated, two additional measures will be used: the Hypersexual Behavior Inventory (HBI)[22] and the Sexual Compulsivity Scale (SCS).[23] Using unpublished data from our research center, the correlations (Pearson's) between HD: CAS and HBI were positive at baseline and post-treatment.
We will also analyze factors (clinical, psychosocial, or biological) that may be predictors of response, and whether there is any difference in participants' readiness to resume pharmacological treatment at end of study.

Furthermore, we will also evaluate tolerability using the UKU side effect rating scale,[24] drug accountability, adherence to treatment, and drop-out rate.

Finally, to aid in understanding CSBD, we will assess for impulsivity, suicidality, and childhood adversities, and collect biological markers (e.g. DNA and hormones) to evaluate their potential association with CSBD. A summary of assessments and biological markers is presented in Table 2, Table 3 and supplement Table 1. Furthermore, an extension of the study is planned including neuroimaging data collection.

Table 3. Study assessments

**Objective**	**Assessments**	**Measure type**	**Time point**
Assess changes in compulsive sexual behaviors from screening to week 8 and follow up	Hypersexual Disorder: Current Assessment Scale (HD: CAS).	Outcome	Baseline, week 2, 4, 6, 8 and end of study
Assess compulsive sexual behaviors	The Hypersexual Disorder Screening Inventory (HDSI)	Eligibility	Baseline, and end of study.

For peer review only - http://bmjopen.bmj.com/site/about/guidelines.xhtml

Assess changes in compulsive sexual behaviors from screening to week 8 and follow up	The Hypersexual Behavior Inventory (HBI)	Outcome	Baseline, week 1-4, week 6, week 8, and end of study
Assess changes in compulsive sexual behaviors from screening to week 8 and follow up	Sexual Compulsivity Scale (SCS)	Outcome	Baseline, week 1-4, week 6, week 8, and end of study
Assess sexual interests	Self-assessment of sexual interests (SSI)	Eligibility	Baseline
Assess sexual dysfunction	International Index of Erectile Function (IIEF)[a]	Eligibility	Baseline
Assess psychiatric co-morbidity	Mini International Neuropsychiatric Interview (MINI)	Eligibility	Baseline
Assess changes in depression severity from screening to week 8 and follow up	Montgomery Åsberg Depression Rating Scale – Self-rating (MADRS-S)	Outcome Safety	Baseline, week 8, and end of study.
Assess change of symptoms of depression and anxiety from screening to week 8 and follow up	Hospital Anxiety and Depression Scale (HAD)	Outcome	Baseline, week 8, and end of study.
Assess changes in alcohol use from screening to week 8 and follow up	Alcohol Use Disorders Identification Test (AUDIT)	Outcome	Baseline, week 8, and end of study.
Assess use of drugs	Drug Use Disorders Identification Test (DUDIT)	Eligibility	Baseline
Assess pathological gambling	Gambling Disorder Identification Test (G-DIT)	Eligibility	Baseline
Assess impulsivity	Barratt Impulsiveness Scale (BIS-11)	Eligibility	Baseline
Assess symptoms of Attention-Deficit/ Hyperactivity Disorder	Adult ADHD Self-Report Scale (ASRS)	Eligibility	Baseline
Assess history of violence	Karolinska Interpersonal Violence Scale (KIVS)	Eligibility	Baseline
Assess history of childhood trauma	The Childhood Trauma Questionnaire – Short Form (CTQ-SF)	Eligibility	Baseline

For peer review only - http://bmjopen.bmj.com/site/about/guidelines.xhtml

Assess suicide ideation and suicidal behavior throughout the study	Columbia Suicide Severity Rating Scale (C-SSRS)	Safety	Baseline, week 1, 4, 8, and end of study.
Assess adverse reactions in week 8	UKU side effect rating scale (UKU)	Tolerability	Week 8
Question of wanting to resume pharmacological treatment, week 14	Yes/No/Don't know option	Outcome	End of study
Notes: [a] assigned men at birth only.			

Adherence and Concomitant Care

Participants can leave the study at any time without giving a reason, however any material already obtained will be analyzed. Participants who miss ≥ 3 doses in a row will be considered as having ceased treatment. Other reasons for discontinuation of treatment include severe adverse reactions, severe psychiatric condition where the safety of the participant or others cannot be guaranteed, initiation of treatment with non-compatible drugs or use of recreational drugs, pregnancy, or start of psychotherapy.

Sample Size and Statistical Methods

Since there is no gold standard for the measurement of pharmacological treatment outcomes in CSBD, the sample size calculation is based on HD: CAS from a study of Cognitive Behavioral Therapy from our clinic[25] (using expanded data available post-publication, n = 76). The difference in HD: CAS pre-post treatment was 4.065 with a standard deviation of 4.74. A statistical power of 80 %, an error probability of 0.05, and a difference between the groups of 3.3 (meaning an effect size of 0.7) would render a sample size of 34 participants in each group. To adjust for potential dropouts we aim to include 80 participants. The results will be analyzed using an appropriate analysis of variance model or a mixed model, depending on the distribution of HD: CAS and the extent of missing data. Participants who discontinue treatment will be included in an intention-to-treat analysis. Per-protocol analysis will also be used. To increase the study's credibility, the extent and nature of missing data will be reported for each treatment group, as well as any predictors of missing data (e.g. young age).

Monitoring

As required, an independent trial investigator from the Karolinska Trial Alliance will regularly control and quality assure the study procedures. There will be no interim analysis.

Patient and public involvement

Patients and the public will not be involved in the design of the study. Advertisement in media will be made.

All subjects will be informed about the study both orally and in writing, including information on the

For peer review only - http://bmjopen.bmj.com/site/about/guidelines.xhtml

potential benefits and risks of the therapies, and informed consent will be collected. The visits at the clinic and the drugs will be free of charge. The same insurance regulation as in ordinary medical care apply. Participants will be informed about the study results on a group level.

Ethics and dissemination

The study procedures will be carried out in accordance with the Declaration of Helsinki and European Good Clinical Practice Guidelines. The Swedish Ethical Review Authority and the Swedish Medical Products Agency have approved the study (Ref. no. 2020-02069 and Ref. no. 5.1-2020-48282). The registration of the trial (Eudra-CT no. 2019-004255-36) is accessible at https://www.clinicaltrialsregister.eu. Study enrollment started in October 2020.

Eligible participants will be aged ≥ 18 years and understand both oral and written Swedish. Paraphilic interests are not an exclusion criterion, whereas severe risk for sexual violence (as reported in the interviews and questionnaires, e.g. participants who report that they actively interact with children for sexual purposes) is.

The study psychiatrists and psychologists are experienced in evaluating and treating patients with CSBD and Paraphilic Disorders, and the investigators will together decide whether the individual may be a potential study candidate.

The risks for the participants including safety aspects of the drugs have been carefully weighed against the potential benefits of conducting the study, and we believe the latter outweighs the former. The participants' health and safety will be assessed and prioritized, which may entail discontinuation of treatment. All undesirable medical events that come to our attention will be graded (mild if the symptoms are transient, moderate if temporary medical treatment for relief is needed, and severe if hospital care or prolonged medical treatment is needed) and followed until the event is resolved. Serious adverse events and suspected unexpected serious adverse reactions are handled and reported as required by the Swedish Medical Products Agency.

Research data from the web-based platform will be stored securely in servers with restricted access. Archived paper material will stored in a locked cabinet, in accordance with current legislation and regulations. Research samples and data will be destroyed 10 years after declaring end of trial to the Swedish Medical Products Agency.

Authorship eligibility will be assured in accordance with The International Committee of Medical Journal Editors (ICMJE) guidelines The results of the study will be presented at scientific conferences and in peer-review articles.

DISCUSSION

For peer review only - http://bmjopen.bmj.com/site/about/guidelines.xhtml

As presented in the introduction, there is currently weak evidence for the pharmacological treatment of CSBD (for review, see Grubbs et al.[26]) and hence a clear need for randomized controlled trials. However, some aspects of this study design and concept need to be discussed:

Feasibility of enrollment

Participants might be unwilling to participate in a drug trial, hence it may be difficult to complete recruitment within the designated time period of three years, leading to a sample too small for rending power in the analyses. In addition, the Covid-19 pandemic might complicate participants' travel to our clinic or even our capacity to conduct the study due to restrictions in the provision of healthcare (e.g. limited to emergency care only). An overrepresentation of participants living in the Stockholm area is to be expected.

Eligibility

We try to minimize the risk for recruiting a homogeneous group of persons with CSBD by inviting individuals with a broad range of severity of symptoms, and the use of randomization with allocation concealment to minimize the risk for selection bias between the groups. Nevertheless, as with most studies, there is a risk for an overrepresentation of those with a better prognosis.

The exclusion criteria are more stringent than the inclusion criteria, partly due to the safety profiles of the drugs – a situation that regardless of study protocol would influence their use in a regular clinical setting. However, we also exclude participants with ongoing recreational drug use to minimize the risk of confounders. We also exclude participants currently being treated with interacting antidepressants and those with a severe psychiatric disorder requiring immediate treatment, as these patients are too sick to follow our study protocol. Most patients seeking treatment at ANOVA for CSBD do not fall into these categories (particularly not the latter), but this exclusion criterion will undoubtedly lead to the inclusion of healthier individuals in the study. Although we aim to have a representative sample, a certain impact on the external validity is to be expected.

Outcome ascertainment

As noted, our main outcome variable HD: CAS is not validated. However, it has a predefined timespan of symptom relief and has been used in previous studies at our research center. When comparing results of HD: CAS with the validated HBI, the latter reaches a floor-effect post treatment, whereas HD: CAS seems to better distinguish effects. Our overall judgement is thus that the advantages of the HD: CAS outweigh the disadvantages.

The HBI has an item addressing craving "My sexual cravings and desires feel stronger than my self-discipline". Nevertheless, we are aware that using this single item to assess craving may be a limitation.

For peer review only - http://bmjopen.bmj.com/site/about/guidelines.xhtml

Another limitation is that this study will not be blinded, and we will not be able to control for the anticipation effect in either group. Nevertheless, group comparison values using regression models with incorporated baseline will still be of major value, and results will nonetheless be of importance to guide clinical praxis and for future meta-analyses. Finally, the study protocol duration of only 14 weeks is a major limitation, but the length chosen for feasibility reasons. Fluoxetine will presumably reach steady state between week four and eight, and with this aspect in mind the comparisons between the groups after researching steady state will be the most informative as well as being deemed sufficient to provide important clinical knowledge.

Acknowledgements

The authors wish to thank Tania McConaghy for language revision.

Contributors

JS, KGÖ, CD and JJ conceived and designed the study. JS and JJ contributed to the statistical plan. JJ is grant holder. JS drafted the manuscript, and all author contributed substantially to refinement of the manuscript. All authors approved the final version.

Funding

This work was supported by the Swedish Research Council grant number 2020-01183 and regional agreements between ANOVA, Region Stockholm, and Region Västerbotten (ALF Region Västerbotten grant number RV-929554). The pharmaceutical companies are not providing funding or being involved in any other way.

Competing interests

Jussi Jokinen has participated in Advisory Board of Janssen concerning esketamine for MDD with current suicidal ideation. The other authors report no conflicts of interest.

Data sharing statement

Individual participant will be available under certain circumstances to researches who provide a methodically sound proposal.

The study protocol and individual participants' data that underlie the results of the published article, after deidentification (text, tables, figures and appendices) will be available to achieve aims in the approved proposal. Data will become available immediately following publication and ending nine years following article publication.

Proposals should be directed to Josephine.savard@umu.se. To gain access, data requestors will need to sign a data access agreement.

For peer review only

Figure 1. Flowchart.

Note: * If unsatisfactory symptom reduction and tolerated.

REFERENCES

1. World Health Organization. The 11th revision of the International Classification of Diseases (ICD-11). Available from: https://icd.who.int/browse11/l-m/en#/http%3a%2f%2fid.who.int%2ficd%2fentity%2f274880002 accessed February 9, 2020.
2. Kuzma JM, Black DW. Epidemiology, prevalence, and natural history of compulsive sexual behavior. *Psychiatr Clin North Am* 2008;31(4):603-11. doi: 10.1016/j.psc.2008.06.005
3. Gola M, Potenza MN. Paroxetine Treatment of Problematic Pornography Use: A Case Series. *Journal of Behavioral Addictions* 2016;5(3):529-32. doi: 10.1556/2006.5.2016.046
4. Winder B, Lievesley R, Elliott H, et al. Evaluation of the use of pharmacological treatment with prisoners experiencing high levels of hypersexual disorder. *J Forens Psychiatry Psychol* 2018;29(1):53-71. doi: 10.1080/14789949.2017.1337801
5. Raymond NC, Grant JE, Kim SW, et al. Treatment of compulsive sexual behaviour with naltrexone and serotonin reuptake inhibitors: two case studies. *Int Clin Psychopharmacol* 2002;17(4):201-5. doi: 10.1097/00004850-200207000-00008
6. Ryback RS. Naltrexone in the treatment of adolescent sexual offenders. *J Clin Psychiatry* 2004;65(7):982-86. doi: 10.4088/JCP.v65n0715
7. Raymond NC, Grant JE, Coleman E. Augmentation with naltrexone to treat compulsive sexual behavior: A case series. *Ann Clin Psychiatry* 2010;22(1):56-62.
8. Bostwick JM, Bucci JA. Internet sex addiction treated with naltrexone. *Mayo Clin Proc* 2008;83(2):226-30. doi: 10.4065/83.2.226
9. Wainberg ML, Muench F, Morgenstern J, et al. A double-blind study of citalopram versus placebo in the treatment of compulsive sexual behaviors in gay and bisexual men. *J Clin Psychiatry* 2006;67(12):1968-73. doi: 10.4088/JCP.v67n1218
10. Kafka MP, Hennen J. The paraphilia-related disorders: an empirical investigation of nonparaphilic hypersexuality disorders in outpatient males. *J Sex Marital Ther* 1999;25(4):305-19. doi: 10.1080/00926239908404008
11. Sutton KS, Stratton N, Pytyck J, et al. Patient Characteristics by Type of Hypersexuality Referral: A Quantitative Chart Review of 115 Consecutive Male Cases. *J Sex Marital Ther* 2015;41(6):563-80. doi: 10.1080/0092623x.2014.935539
12. Kafka MP, Hennen J. Hypersexual desire in males: are males with paraphilias different from males with paraphilia-related disorders? *Sex Abuse* 2003;15(4):307-21. doi: 10.1177/107906320301500407
13. Engel J, Veit M, Sinke C, et al. Same Same but Different: A Clinical Characterization of Men with Hypersexual Disorder in the Sex@Brain Study. *J Clin Med* 2019;8(2) doi: 10.3390/jcm8020157
14. Thibaut F, De La Barra F, Gordon H, et al. The World Federation of Societies of Biological Psychiatry (WFSBP) guidelines for the biological treatment of paraphilias. *World J Biol Psychiatry* 2010;11(4):604-55. doi: 10.3109/15622971003671628
15. Thibaut F, Cosyns P, Fedoroff JP, et al. The World Federation of Societies of Biological Psychiatry (WFSBP) 2020 guidelines for the pharmacological treatment of paraphilic disorders. *The World Journal of Biological Psychiatry* 2020;21(6):412-90. doi: 10.1080/15622975.2020.1744723
16. Franck J, Jayaram-Lindstrom N. Pharmacotherapy for alcohol dependence: status of current treatments. *Curr Opin Neurobiol* 2013;23(4):692-9. doi: 10.1016/j.conb.2013.05.005
17. Savard J, Öberg KG, Chatzittofis A, et al. Naltrexone in Compulsive Sexual Behavior Disorder: A Feasibility Study of Twenty Men. *J Sex Med* 2020 doi: 10.1016/j.jsxm.2020.04.318

For peer review only - http://bmjopen.bmj.com/site/about/guidelines.xhtml

18. Sheehan DV, Lecrubier Y, Sheehan KH, et al. The Mini-International Neuropsychiatric Interview (M.I.N.I.): the development and validation of a structured diagnostic psychiatric interview for DSM-IV and ICD-10. *J Clin Psychiatry* 1998;59 Suppl 20:22-33;quiz 34-57.
19. Posner K, Brown GK, Stanley B, et al. The Columbia–Suicide Severity Rating Scale: Initial Validity and Internal Consistency Findings From Three Multisite Studies With Adolescents and Adults. *Am J Psychiatry* 2011;168(12):1266-77. doi: 10.1176/appi.ajp.2011.10111704
20. Kafka MP. Hypersexual disorder: a proposed diagnosis for DSM-V. *Arch Sex Behav* 2010;39(2):377-400. doi: 10.1007/s10508-009-9574-7 [published Online First: 2009/11/26]
21. American Psychiatric Association. Hypersexual Disorder: current assessment scale. American Psychatric Association's DSM-V workgroup on Sexual and Gender Identity Disorders 2010. Available from: http://www.dsm5.org/ProposedRevisions/Pages/proposedrevision.aspx?rid=415 accessed March 2010.
22. Reid RC, Garos S, Carpenter BN. Reliability, Validity, and Psychometric Development of the Hypersexual Behavior Inventory in an Outpatient Sample of Men. *Sexual Addiction & Compulsivity* 2011;18(1):30-51. doi: 10.1080/10720162.2011.555709
23. Kalichman SC, Rompa D. Sexual sensation seeking and Sexual Compulsivity Scales: reliability, validity, and predicting HIV risk behavior. *J Pers Assess* 1995;65(3):586-601. doi: 10.1207/s15327752jpa6503_16
24. Lindstrom E, Lewander T, Malm U, et al. Patient-rated versus clinician-rated side effects of drug treatment in schizophrenia. Clinical validation of a self-rating version of the UKU Side Effect Rating Scale (UKU-SERS-Pat). *Nord J Psychiatry* 2001;55 Suppl 44:5-69. doi: 10.1080/080394801317084428
25. Hallberg J, Kaldo V, Arver S, et al. Internet-Administered Cognitive Behavioral Therapy for Hypersexual Disorder, With or Without Paraphilia(s) or Paraphilic Disorder(s) in Men: A Pilot Study. *J Sex Med* 2020;17(10):2039-54. doi: 10.1016/j.jsxm.2020.07.018
26. Grubbs JB, Hoagland KC, Lee BN, et al. Sexual addiction 25 years on: A systematic and methodological review of empirical literature and an agenda for future research. *Clin Psychol Rev* 2020;82:101925. doi: https://doi.org/10.1016/j.cpr.2020.101925

For peer review only

For peer review only - http://bmjopen.bmj.com/site/about/guidelines.xhtml

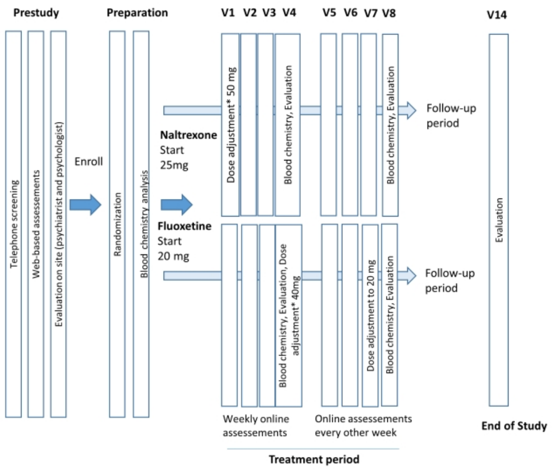

Flowchart.

338x190mm (300 x 300 DPI)

Supplementary Table 1
Outcome Measures with Psychometric Properties

Name	Psychometric properties
The Mini International Neuropsychiatric Interview (MINI)	Validated<br>(Sheehan et al., 1998).
Hypersexual Disorder: Current Assessment Scale (HD:CAS)	Not validated<br>Cronbach's α = 0.76<br>(Savard., 2021)
The Hypersexual Behavior Inventory (HBI)	Validated<br>test-retest reliability r = 0.91; Cronbach's α = 0.96<br>(Reid et al., 2011)
Self-assessment of Sexual Interest (SSI)	Not validated<br>(Långström, unpublished)
The Hypersexual Disorder Screening Inventory (HDSI)	Validated in Swedish<br>inter-rater reliability r = 0.51, Cronbach's α = 0.80-0.81<br>(Öberg et al., 2017)
International Index of Erectile Function (IIEF)	Validated<br>test–retest reliability r = 0.64–0.84; Cronbach's α = 0.73–0.99) across studies.<br>(Rosen et al., 1997; 2002)
Sexual Compulsivity Scale (SCS)	Cronbach's α = 0.89-0.92<br>(Kalichman & Rompa, 1995)
The Alcohol Use Disorders Identification Test (AUDIT)	Validated in Swedish<br>test-retest reliability r = 0.97; Cronbach's α 0.82<br>(Bergman & Kallmen, 2002)
The Drug Use Disorders Identification Test (DUDIT)	Validated in Swedish<br>Cronbach's α = 0.80; sensitivity (ranging from 0.85- 1.00) and specificity (ranging from 0.75 to 0.92)<br>(Berman et al., 2005; Hildebrand 2015)
The Gambling Disorder Identification Test (G-DIT)	Test-retest reliability<br>intraclass correlation coefficient = 0.93<br>Cronbach's α = 0.94<br>(Molander et. al., 2021)
The Childhood Trauma Questionnaire – Short Form (CTQ-SF)	Validated in Swedish<br>The inter-correlations between CTQ total scale and subscales vary between r = 0.15-0.89; Cronbach's α = 0.92 on the total score, subscales α = 0.65-0.86<br>(Bernstein & Fink, 1998; Gerdner & Allgulander 2009)

For peer review only

Karolinska Interpersonal Violence Scale (KIVS)	Validated in Swedish<br>inter-rater reliability for the subscales *r* = 0.91-0.95<br>(Jokinen et al., 2010)
Adult ADHD Self-Report Scale (ASRS)	Validated<br>test-retest reliability *r* = 0.58-0.77<br>(Kessler et al., 2007)
The Barratt Impulsiveness Scale (BIS)	Validated<br>test-retest reliability Spearman's rho = 0.83<br>Cronbach's *α* = 0.83<br>(Stanford et al., 2009)
Montgomery Åsberg Depression Rating Scale – Self-rating (MADRS-S)	Validated in Swedish<br>inter-rater reliability *r* = 0.87; Cronbach's α = 0.84<br>(Svanborg & Asberg 2001; Fantino, B., & Moore, N. 2009)
Hospital Anxiety and Depression Scale (HAD)	Validated in Swedish<br>test-retest reliability *r* = 0.72; Cronbachs α = 0.89-0.93<br>(Lisspers, J., et al. 1997)
Columbia Suicide Severity Rating Scale (C-SSRS)	Validated in Swedish<br>C-SSRS total score, AUC = 0.65 and a cut-off of 28.5 gave a sensitivity of 69% and a specificity of 54% in predicting a non-fatal or fatal suicide attempt<br>Cronbach's α = 0.73-0.95<br>(Posner et al., 2011; Lindh et al., 2018)
UKU side effect rating scale (UKU)	Validated<br>inter-rater reliability *r* = 0.07-0.80<br>(Lingjaerde et al., 1987; Lindström 2001)

Notes: Bergman, H., & Kallmen H. (2002). Alcohol use among Swedes and a psychometric evaluation of the alcohol use disorders identification test. Alcohol and Alcoholism, 37(3), 245–251. https://doi.org/10.1093/alcalc/37.3.245

Berman, A. H., Bergman, H., Palmstierna, T., & Schlyter, F. (2005). Evaluation of the Drug Use Disorders Identification Test (DUDIT) in Criminal Justice and Detoxification Settings and in a Swedish Population Sample. European Addiction Research, 11(1), 22-31. https://doi.org/10.1159/000081413

Bernstein DP, Fink L. Childhood Trauma Questionnaire. A retrospective self-report. Manual. The Psychological Corporation, Harcourt Brace & Company, San Antonio, TX 1998

Fantino, B., & Moore, N. (2009). The self-reported Montgomery-Asberg Depression Rating Scale is a useful evaluative tool in Major Depressive Disorder. BMC psychiatry, 9, 26. https://doi.org/10.1186/1471-244X-9-26

Gerdner, A., & Allgulander, C. (2009). Psychometric properties of the Swedish version of the Childhood Trauma Questionnaire-Short Form (CTQ-SF). Nordic journal of psychiatry, 63(2), 160–170. https://doi.org/10.1080/08039480802514366

Hildebrand M. (2015). The Psychometric Properties of the Drug Use Disorders Identification Test (DUDIT): A Review of Recent Research. Journal of substance abuse treatment, 53, 52–59. https://doi.org/10.1016/j.jsat.2015.01.008

Jokinen, J., Forslund, K., Ahnemark, E., Gustavsson, J. P., Nordström, P., & Asberg, M. (2010). Karolinska Interpersonal Violence Scale predicts suicide in suicide attempters. The Journal of clinical psychiatry, 71(8), 1025–1032. https://doi.org/10.4088/JCP.09m05944blu

Kalichman, S. C., & Rompa, D. (1995). Sexual sensation seeking and Sexual Compulsivity Scales: reliability, validity, and predicting HIV risk behavior. Journal of personality assessment, 65(3), 586–601. https://doi.org/10.1207/s15327752jpa6503_16

Kessler, R. C., Adler, L. A., Gruber, M. J., Sarawate, C. A., Spencer, T., & Van Brunt, D. L. (2007). Validity of the World Health Organization Adult ADHD Self-Report Scale (ASRS) Screener in a representative sample of health plan members. International journal of methods in psychiatric research, 16(2), 52–65. https://doi.org/10.1002/mpr.208

Lindh, Å.U., Waern, M., Beckman, K. et al. Short term risk of non-fatal and fatal suicidal behaviours: the predictive validity of the Columbia-Suicide Severity Rating Scale in a Swedish adult psychiatric population with a recent episode of self-harm. BMC Psychiatry 18, 319 (2018). https://doi.org/10.1186/s12888-018-1883-8

Lindström, E., Lewander, T., Malm, U., Malt, U. F., Lublin, H., & Ahlfors, U. G. (2001). Patient-rated versus clinician-rated side effects of drug treatment in schizophrenia. Clinical validation of a self-rating version of the UKU Side Effect Rating Scale (UKU-SERS-Pat). Nordic journal of psychiatry, 55 Suppl 44, 5–69. https://doi.org/10.1080/080394801317084428

Lingjaerde, O., Ahlfors, U. G., Bech, P., Dencker, S. J., & Elgen, K. (1987). The UKU side effect rating scale. A new comprehensive rating scale for psychotropic drugs and a cross-sectional study of side effects in neuroleptic-treated patients. Acta psychiatrica Scandinavica. Supplementum, 334, 1–100. https://doi.org/10.1111/j.1600-0447.1987.tb10566.x

Lisspers, J., Nygren, A., & Söderman, E. (1997). Hospital Anxiety and Depression Scale (HAD): some psychometric data for a Swedish sample. Acta psychiatrica Scandinavica, 96(4), 281–286. https://doi.org/10.1111/j.1600-0447.1997.tb10164.x

Långström N. Self-assessment of Sexual Interest (SSI). Unpublished questionnaire. 2010.

Molander, O., Wennberg, P., & Berman, A. H. (2021). The Gambling Disorders Identification Test (GDIT): Psychometric Evaluation of a New Comprehensive Measure for Gambling Disorder and Problem Gambling. Assessment, 10731911211046045. Advance online publication. https://doi.org/10.1177/10731911211046045

Posner, K., Brown, G. K., Stanley, B., Brent, D. A., Yershova, K. V., Oquendo, M. A., Currier, G. W., Melvin, G. A., Greenhill, L., Shen, S., & Mann, J. J. (2011). The Columbia-Suicide Severity Rating Scale: initial validity and internal consistency findings from three multisite studies with adolescents and adults. The American journal of psychiatry, 168(12), 1266–1277. https://doi.org/10.1176/appi.ajp.2011.10111704

Reid, R. C., Garos, S., & Carpenter, B. N. (2011). Reliability, Validity, and Psychometric Development of the Hypersexual Behavior Inventory in an Outpatient Sample of Men. Sexual Addiction & Compulsivity, 18(1), 30-51. https.//doi.org/10.1080/10720162.2011.555709

For peer review only

Rosen, R. C., Riley, A., Wagner, G., Osterloh, I. H., Kirkpatrick, J., & Mishra, A. (1997). The international index of erectile function (IIEF): a multidimensional scale for assessment of erectile dysfunction. Urology, 49(6), 822–830. https://doi.org/10.1016/s0090-4295(97)00238-0

Rosen, R. C., Cappelleri, J. C., & Gendrano, N., 3rd (2002). The International Index of Erectile Function (IIEF): a state-of-the-science review. International journal of impotence research, 14(4), 226–244. https://doi.org/10.1038/sj.ijir.3900857

Savard, J. (2021). Compulsive sexual behavior disorder: clinical characteristics and treatment with Naltrexone (PhD dissertation, Umeå universitetet). Retrieved from http://urn.kb.se/resolve?urn=urn:nbn:se:umu:diva-186811

Sheehan, D. V., Lecrubier, Y., Sheehan, K. H., Amorim, P., Janavs, J., Weiller, E., Hergueta, T., Baker, R., & Dunbar, G. C. (1998). The Mini-International Neuropsychiatric Interview (M.I.N.I.): the development and validation of a structured diagnostic psychiatric interview for DSM-IV and ICD-10. Journal of Clinical Psychiatry, 59 Suppl 20, 22-33;quiz 34-57

Stanford, M. S., Mathias, C. W., Dougherty, D. M., Lake, S. L., Anderson, N. E., & Patton, J. H. (2009). Fifty years of the Barratt Impulsiveness Scale: An update and review. Personality and Individual Differences, 47(5), 385–395. https://doi.org/10.1016/j.paid.2009.04.008

Svanborg P, Asberg M. A comparison between the Beck Depression Inventory (BDI) and the self-rating version of the Montgomery Asberg Depression Rating Scale (MADRS). J Affect Disord. 2001;64(2-3):203-16.

Öberg, K. G., Hallberg, J., Kaldo, V., Dhejne, C., & Arver, S. (2017). Hypersexual Disorder According to the Hypersexual Disorder Screening Inventory in Help-Seeking Swedish Men and Women With Self-Identified Hypersexual Behavior. Sexual medicine, 5(4), e229–e236. https://doi.org/10.1016/j.esxm.2017.08.001

For peer review only

STANDARD PROTOCOL ITEMS: RECOMMENDATIONS FOR INTERVENTIONAL TRIALS

SPIRIT 2013 Checklist: Recommended items to address in a clinical trial protocol and related documents*

Section/item	Item No	Description	Addressed on page number
**Administrative information**			
Title	1	Descriptive title identifying the study design, population, interventions, and, if applicable, trial acronym	Title page
Trial registration	2a	Trial identifier and registry name. If not yet registered, name of intended registry	Abstract
	2b	All items from the World Health Organization Trial Registration Data Set	Link in abstract
Protocol version	3	Date and version identifier	4
Funding	4	Sources and types of financial, material, and other support	14
Roles and responsibilities	5a	Names, affiliations, and roles of protocol contributors	Title page
	5b	Name and contact information for the trial sponsor	Title page
	5c	Role of study sponsor and funders, if any, in study design; collection, management, analysis, and interpretation of data; writing of the report; and the decision to submit the report for publication, including whether they will have ultimate authority over any of these activities	14
	5d	Composition, roles, and responsibilities of the coordinating centre, steering committee, endpoint adjudication committee, data management team, and other individuals or groups overseeing the trial, if applicable (see Item 21a for data monitoring committee)	11

For peer review only

**Introduction**			
Background and rationale	6a	Description of research question and justification for undertaking the trial, including summary of relevant studies (published and unpublished) examining benefits and harms for each intervention	2-3
	6b	Explanation for choice of comparators	3
Objectives	7	Specific objectives or hypotheses	3-4
Trial design	8	Description of trial design including type of trial (eg, parallel group, crossover, factorial, single group), allocation ratio, and framework (eg, superiority, equivalence, noninferiority, exploratory)	4
**Methods: Participants, interventions, and outcomes**			
Study setting	9	Description of study settings (eg, community clinic, academic hospital) and list of countries where data will be collected. Reference to where list of study sites can be obtained	4
Eligibility criteria	10	Inclusion and exclusion criteria for participants. If applicable, eligibility criteria for study centres and individuals who will perform the interventions (eg, surgeons, psychotherapists)	5
Interventions	11a	Interventions for each group with sufficient detail to allow replication, including how and when they will be administered	6-8 + figure 1
	11b	Criteria for discontinuing or modifying allocated interventions for a given trial participant (eg, drug dose change in response to harms, participant request, or improving/worsening disease)	11
	11c	Strategies to improve adherence to intervention protocols, and any procedures for monitoring adherence (eg, drug tablet return, laboratory tests)	9
	11d	Relevant concomitant care and interventions that are permitted or prohibited during the trial	11
Outcomes	12	Primary, secondary, and other outcomes, including the specific measurement variable (eg, systolic blood pressure), analysis metric (eg, change from baseline, final value, time to event), method of aggregation (eg, median, proportion), and time point for each outcome. Explanation of the clinical relevance of chosen efficacy and harm outcomes is strongly recommended	9 + Table 3
Participant timeline	13	Time schedule of enrolment, interventions (including any run-ins and washouts), assessments, and visits for participants. A schematic diagram is highly recommended (see Figure)	Figure 1

For peer review only - http://bmjopen.bmj.com/site/about/guidelines.xhtml

Sample size	14	Estimated number of participants needed to achieve study objectives and how it was determined, including clinical and statistical assumptions supporting any sample size calculations	11
Recruitment	15	Strategies for achieving adequate participant enrolment to reach target sample size	6, 11
**Methods: Assignment of interventions (for controlled trials)**			
Allocation:			
Sequence generation	16a	Method of generating the allocation sequence (eg, computer-generated random numbers), and list of any factors for stratification. To reduce predictability of a random sequence, details of any planned restriction (eg, blocking) should be provided in a separate document that is unavailable to those who enrol participants or assign interventions	8
Allocation concealment mechanism	16b	Mechanism of implementing the allocation sequence (eg, central telephone; sequentially numbered, opaque, sealed envelopes), describing any steps to conceal the sequence until interventions are assigned	8
Implementation	16c	Who will generate the allocation sequence, who will enrol participants, and who will assign participants to interventions	8
Blinding (masking)	17a	Who will be blinded after assignment to interventions (eg, trial participants, care providers, outcome assessors, data analysts), and how	N/A
	17b	If blinded, circumstances under which unblinding is permissible, and procedure for revealing a participant's allocated intervention during the trial	N/A
**Methods: Data collection, management, and analysis**			
Data collection methods	18a	Plans for assessment and collection of outcome, baseline, and other trial data, including any related processes to promote data quality (eg, duplicate measurements, training of assessors) and a description of study instruments (eg, questionnaires, laboratory tests) along with their reliability and validity, if known. Reference to where data collection forms can be found, if not in the protocol	6, 9, Table 3, supplement table 1
	18b	Plans to promote participant retention and complete follow-up, including list of any outcome data to be collected for participants who discontinue or deviate from intervention protocols	11

Data management	19	Plans for data entry, coding, security, and storage, including any related processes to promote data quality (eg, double data entry; range checks for data values). Reference to where details of data management procedures can be found, if not in the protocol	12
Statistical methods	20a	Statistical methods for analysing primary and secondary outcomes. Reference to where other details of the statistical analysis plan can be found, if not in the protocol	11
	20b	Methods for any additional analyses (eg, subgroup and adjusted analyses)	11
	20c	Definition of analysis population relating to protocol non-adherence (eg, as randomised analysis), and any statistical methods to handle missing data (eg, multiple imputation)	11
**Methods: Monitoring**			
Data monitoring	21a	Composition of data monitoring committee (DMC); summary of its role and reporting structure; statement of whether it is independent from the sponsor and competing interests; and reference to where further details about its charter can be found, if not in the protocol. Alternatively, an explanation of why a DMC is not needed	11
	21b	Description of any interim analyses and stopping guidelines, including who will have access to these interim results and make the final decision to terminate the trial	11
Harms	22	Plans for collecting, assessing, reporting, and managing solicited and spontaneously reported adverse events and other unintended effects of trial interventions or trial conduct	8, 9, 11, 12
Auditing	23	Frequency and procedures for auditing trial conduct, if any, and whether the process will be independent from investigators and the sponsor	11
**Ethics and dissemination**			
Research ethics approval	24	Plans for seeking research ethics committee/institutional review board (REC/IRB) approval	12
Protocol amendments	25	Plans for communicating important protocol modifications (eg, changes to eligibility criteria, outcomes, analyses) to relevant parties (eg, investigators, REC/IRBs, trial participants, trial registries, journals, regulators)	4, 12

Consent or assent	26a	Who will obtain informed consent or assent from potential trial participants or authorised surrogates, and how (see Item 32)	11
	26b	Additional consent provisions for collection and use of participant data and biological specimens in ancillary studies, if applicable	Supplement material (Patient consent form)
Confidentiality	27	How personal information about potential and enrolled participants will be collected, shared, and maintained in order to protect confidentiality before, during, and after the trial	12, 14
Declaration of interests	28	Financial and other competing interests for principal investigators for the overall trial and each study site	14
Access to data	29	Statement of who will have access to the final trial dataset, and disclosure of contractual agreements that limit such access for investigators	14
Ancillary and post-trial care	30	Provisions, if any, for ancillary and post-trial care, and for compensation to those who suffer harm from trial participation	11-12
Dissemination policy	31a	Plans for investigators and sponsor to communicate trial results to participants, healthcare professionals, the public, and other relevant groups (eg, via publication, reporting in results databases, or other data sharing arrangements), including any publication restrictions	12
	31b	Authorship eligibility guidelines and any intended use of professional writers	12
	31c	Plans, if any, for granting public access to the full protocol, participant-level dataset, and statistical code	14
**Appendices**			
Informed consent materials	32	Model consent form and other related documentation given to participants and authorised surrogates	Supplement material (patient consent form)
Biological specimens	33	Plans for collection, laboratory evaluation, and storage of biological specimens for genetic or molecular analysis in the current trial and for future use in ancillary studies, if applicable	Supplement material (patient consent form)

For peer review only - http://bmjopen.bmj.com/site/about/guidelines.xhtml

*It is strongly recommended that this checklist be read in conjunction with the SPIRIT 2013 Explanation & Elaboration for important clarification on the items. Amendments to the protocol should be tracked and dated. The SPIRIT checklist is copyrighted by the SPIRIT Group under the Creative Commons "Attribution-NonCommercial-NoDerivs 3.0 Unported" license.

For peer review only

INFORMED CONSENT FORM

Pharmacological treatement of Compulsive Sexual Behavior

- a comparison of the effectiveness of naltrexon versus fluoxetin: the CeSar-study

Background and aim

Here at ANOVA clinic we treat persons who experience a troublesome or dangerous sexuality. There is a lack of knowledge regarding treatment of intrusive sexual thoughts, impulses and behaviors in individuals with compulsive sexuality, sometimes called "sexual addiction". This study is evaluating a new drug for this purpose, naltrexone. Naltrexone is currently used in the treatment of alcohol dependence and has been reported as a drug that can also reduce both urges and behaviors linked to compulsive sexual behavior. To compare the new treatment, half of the participants will instead receive fluoxetine which is used for the treatment of compulsive sexuality nationally and internationally.
The overall purpose of this study is to improve the treatment options and the quality of life for the individual.

Request for participation

You have contacted or been referred to ANOVA and are therefore asked to participate. Participation is voluntary.

How is the study conducted?

If you are interested in participating in the study and provide an informed consent in the web platform, you will be asked to complete an online survey for approximately 60-90 minutes with questions on your sexuality and your general well-being. The online survey is the first step in the study and aims to provide increased knowledge about sexuality that leads to negative consequences.
After you have filled in the online survey, you will be contacted by mail or telephone and offered an appointment at the clinic ANOVA. You will meet a physician and psychologist for two interviews. They will evaluate if you meet the requirements to be enrolled in the study and you will have the opportunity to ask questions. If you want to proceed with the study, you will be requested to sign an informed consent for participation in the drug treatment. You will also be asked to participate in a computerized test of impulsivity and to submit a routine blood test to map sex hormones and liver-, thyroid- and kidney function and metabolic status (= 5 tubes, approximately 20ml) before treatment. We also ask for a urine sample to have objective measures to exclude a recreational drug use. This urine sample is discarded if it is negative or sent to a laboratory for further analysis if positive as done with routine sample in healthcare. If you are a woman of childbearing age, you will have to submit a sample (urine or blood sample, a 3.5ml tube) to rule out pregnancy and are encouraged to use a safe contraception method during the study.

For peer review only - http://bmjopen.bmj.com/site/about/guidelines.xhtml

We also ask if you are willing to provide research blood samples (max. 100ml) with the aim to examine neurobiological markers of compulsive sexuality. These samples include e.g. DNA extraction, see further under the heading Biobank below.

Estimated time required for the visits is about 3 hours.

If you are offered treatment within the framework of the study, you will receive treatment for 8 weeks with either naltrexone or fluoxetine. The selection is made through randomization (i.e. assignment by chance). You will know which of the preparations you have been randomized to, but will not have the opportunity to choose drug.

You will be contacted by phone after 3-5 days and after 4 weeks to inquire about mood and possibly adjust the dose. At 4 weeks, you will be asked to provide new blood samples (3 tubes, approx. 12 ml).
You will be asked to report any side effects and if you notice any changes since the start of medication in the web-based platform. In addition, you will be asked to fill questionnaires every week that assess you mood and your sexual problems. Filling in questionnaires will take about 10-20 minutes per week.
You will be contacted and offered a psychologist or physicians' appointment if we become concerned about your mental or physical state.

Visit 2 at ANOVA: After finishing treatment, you will have a follow-up visit with a physician. You will also be asked to provide urine / blood to see the concentration of the drug in the body, routine blood tests (4 tubes, approx. 16 ml) and research blood samples (2 tubes, approx. 8 ml) to examine whether the treatment has i.e. affected hormone levels.

Visit 3 at ANOVA: Six weeks after the end of treatment, you have a final appointment with a physician who follows up on your mood and your sexuality.

Visits 2 and 3 take about 30-60 minutes each.

If you need care after the trail, you will be guided to the type of care that best suits you. Regardless of whether you are offered treatment or not, your answers in the online survey will be an important part of the study.

Biobank

Routine blood samples will be handled and destroyed according to the hospital's guidelines as in normal sampling in healthcare. The samples that are intended to be stored (5 tubes, max 30 ml) for e.g. DNA analysis and measurement of the hormone oxytocin will be stored in a biobank. The responsible biobank is Stockholms Medicinska Biobank (reg. No. 914) in accordance with the Act on Biobanks in Health Care (2002: 297), which regulates the manner

For peer review only

in which samples may be saved and used, and it also regulates the quality and safety of biobanks.
The samples will be stored coded, which means that the samples cannot be directly traced to you as a person. The samples and the associated identification list (code key) will be kept separate from each other, and protected from access by unauthorized persons.

Coded samples may be sent for analysis both within Sweden and the EU / USA.

Future projects: The blood samples will be stored pending analysis for up to 10 years, after which they will be destroyed. The samples may also be relevant for not yet unplanned research projects if you approve storage for this. A new ethical application will be made in such case, and you may be contacted again with a request for your consent. A separate request regarding future projects is provided in the enclosed consent form.

If you regret that you gave permission for your sample to be stored, you have the right to have the samples destroyed or deidentified by contacting test leader Josephine Savard, please find contact information below.

Are there any risks associated with the study?

Answering questions on private topics such as sexuality and mental health might feel uncomfortable, but these questions need to be asked so we can help you.
As for the medicine, you can experience side effects that are usually transient such as nausea, headache, vomiting and weakness. Serious side effects are uncommon with both drugs. For safety reasons, you will have to provide blood samples before and after the treatment to map, among other things, liver function. Should your samples be abnormal, we will process it according to medical practice. If you are a woman of reproductive age, you need to use safe contraception throughout the study period and submit a negative pregnancy test to be included in the study.

While blood sampling, you may experience temporary discomfort, dizziness and you may get a bruise.

During the course of the study, you could use drugs temporary such as painkillers (e.g. paracetamol) or short-term anti-anxiety medication (e.g. Atarax). Other treatment should be avoided and may lead to discontinuation from the study. This specially applies to drugs with addictive features. Ongoing drug treatment is usually suitable to continue with, but dose changes should be avoided. If in doubt, discuss with your treating physician at ANOVA.

Warning # 1! Should you develop symptoms such as jaundice / hepatitis (inflammation of the liver), you must immediately contact an emergency department or call 112. Symptoms of jaundice are feelings of illness, yellow staining of the skin and whites of the eyes, abdominal pain, light stools and yellow urine.

For peer review only

Warning # 2! Naltrexone counteracts the effect of opioids found in e.g. painkillers (Citodon, OxyContin /OxyNorm, Dolcontin, morphine, etc.) and in cough medicines (Cocillana-Etyfin). There are also opioids in loperamide (Dimor, Loperamide, Imodium) used to treat diarrhea. Should an emergency situation arise where you need treatment with opioids, you should immediately stop taking the study drug and inform the responsible physician about the simultaneous participation in this study.

Warning # 3! Should you during the course of treatment deteriorate mentally with e.g. suicidal ideation, you must contact the study physiatrist or psychiatric emergency service at your place of residence. You will receive contact information at the start of study.

We recommend that you always carry the plastic card you are assigned at start of the study. The card has contact information to the study supervisor so that the treatment unit can obtain information about the study

Are there any benefits to participating in the study?

There is a possibility that you will feel relieved to have shared your thoughts and feelings with a professional and that the drug takes the edge off the desire to commit sexual acts. It can also be an advantage to have contacted our clinic to enroll as a "regular patient" after the study period is over.

Data management and confidentiality

Information about you will be registered within the framework of the study:
- Self-assessments are handled in a web-based platform that is encrypted and protected with double authentication requirements on a secure server at Karolinska Institutet.
- Other data such as interview forms and biobank samples will be handled pseudonymised, i.e. linked to you through a study code. The code key will be kept at ANOVA and protected from access by unauthorized persons.

Your answers and results will be stored in locked folders, secure digital files, encrypted databases and servers in accordance with the General Data Protection Regulation (GDPR 2016/619). Your information will not be used commercially and will be stored for a maximum of 10 years after the end of the study. All information will be handled with total confidentiality. It will not be possible to link any information to you as an individual when the results are reported.

As in ANOVA's regular care, computer-based patient records will be generated. These will not be visible outside ANOVA's record domain.

In order to verify that the collected data is handled correct, and that the procedures are performed in accordance with applicable laws and regulations, the implementation of the research study will be reviewed by an independent trial investigator. Investigators who review

For peer review only

the research study may be given access to decoded data and patient records together with your responsible physician and under confidentiality.

Personal data is protected and processed in accordance with the General Data Protection Regulation (GDPR 2016/679) and the Public Access to Information and Secrecy Act (2009: 400). No unauthorized person will be allowed to access the information. According to the General Data Protection Regulation, you have the right to obtain the information that is handled of you in the study free of charge and, if necessary, have any errors corrected.

If you want to take part of the information, you can contact test leader Dr Josephine Savard for more information, contact information can be found on the last page under the section Responsible. Responsible for the management of personal data is Karolinska University Hospital's Data Protection Office. The Data Protection Officer can be reached at: Karolinska University Hospital, 171 76 Solna, tel. 08-517 700 00 (switchboard operator), e-mail: dataskyddsombud.karolinska@regionstockholm.se. If you are dissatisfied with how your personal data is processed, you have the right to submit a complaint to the Swedish Authority for Privacy Protection, which is the supervisory authority.

How do I get information about the results of the study?

The results will be published in scientific journals. Only statistical variables will be presented, and it will not be possible to identify individual participants. If you want to take part of the scientific reports, you are welcome to contact ANOVA where this study will be carried out. Information on clinical trials can be found at: www.clinicaltrials.gov

Insurance and compensation

The regular patient insurance applies. The drugs and the visits will be free of charge.

Participation is voluntary

Participation in the study is voluntary and you can withdraw your participation at any time without stating why and without it affecting your future care at ANOVA. If you want to discontinue your participation, contact the study supervisor or the test leader (contact information below). After you withdraw your participation, no new information about you will be saved. Already collected data will be preserved.
Samples: If you regret that you gave permission for us to store your samples, you have the right to have the samples discarded (this means that the samples will be destroyed or deidentified).

Responsible for the trial

Responsible for the implementation of the study is ANOVA, Karolinska University Hospital. The principal researcher is Professor Jussi Jokinen (jussi.jokinen@ki.se)

For peer review only

For questions, please contact test leader Josephine Savard, research nurse Susanne Jarlvik Alm or research assistant Pia Jaensson

Dr. Josephine Savard (Testleader)
ANOVA, Karolinska Universitetssjukhuset, 171 76 Stockholm.
E-post: josephine.savard@regionstockholm.se, Tel +46 (0)72-5823241

Susanne Jarlvik Alm
ANOVA, Karolinska Universitetssjukhuset, 171 76 Stockholm.
E-post: Susanne.jarlvik-alm@regionstockholm.se, Tel +46 (0) 8-51772935

Pia Jaensson
ANOVA, Karolinska Universitetssjukhuset, 171 76 Stockholm.
E-post: pia.jaensson@regionstockholm.se, Tel +46 (0) 8-51773832

For peer review only

Consent form: Drug treatment for compulsive sexuality, CeSar study.

• I have read the written information regarding the research study and I have had the opportunity to ask questions and have them answered.
• I provide my consent to participate in the study and know that my participation is completely voluntary. I am aware that blood and urine samples need to be provided based on clinical indication. I regulate how the samples are saved (see below).
• I am aware that I can withdraw my consent and terminate my participation at any time and without explanation, as well as having collected samples discarded. The samples will then be destroyed or deidentified.
• I allow my personal information to be registered according to the information I have received and that collected data about me is stored and handled electronically by study supervisors. I allow unidentified data to be analyzed in future, unplanned studies after approval by the Ethics Review Authority.
• I allow the study supervisor or a study monitor to receive patient record information that is relevant to the current research study and that the Swedish Medical Products Agency and the corresponding supervisory authority within and outside the EU receive patient record information during any supervision.

Signature Name in block letters Date

---------------------------- -------------------------------- -------------------

Consent, research on blood samples within the framework of the study: I give my consent that the samples I submit will be saved in the biobank and that the samples are used for research in accordance with what is described in the patient consent information.

Yes □ No □

Consent, preservation of samples for future research studies: Your samples may be valuable for unplanned research projects (as described in this information) if you approve storage for this. In these cases, a new ethical review will take place and you may be contacted again with a request. Do you consent to that samples that remain after the completion of the Cesar study are stored for future research and may be analyzed within Sweden, the EU and the USA?

Yes □ No □
Signature Name in block letters Date

------------------------------ -------------------------------- -------------------

To be completed by the investigator: I confirm that I have provided both oral and written information about the described trial and that a copy of the patient information sheet has been handed out to the patient.

Signature Name in block letters Date

------------------------------ ---------------------------------- -------------------

For peer review only

BMJ Open

A Randomized Controlled Trial of Fluoxetine Versus Naltrexone in Compulsive Sexual Behavior Disorder: Presentation of the Study Protocol

Journal:	*BMJ Open*
Manuscript ID	bmjopen-2021-051756.R2
Article Type:	Protocol
Date Submitted by the Author:	26-Apr-2022
Complete List of Authors:	Savard, Josephine; Umea University, Department of Clinical Sciences/psychiatry; Karolinska University Hospital, ANOVA<br>Görts Öberg, Katarina; Karolinska University Hospital, ANOVA; Karolinska Institute, Department of Medicine<br>Dhejne, Cecilia; Karolinska University Hospital, ANOVA; Karolinska Institute, Department of Medicine<br>Jokinen, Jussi; Umea University, Department of Clinical Sciences/psychiatry; Karolinska Institute, Centre for Psychiatry Research, Department of Clinical Neuroscience
**Primary Subject Heading**:	Mental health
Secondary Subject Heading:	Sexual health
Keywords:	Impulse control disorders < PSYCHIATRY, Sexual and gender disorders < PSYCHIATRY, Adult psychiatry < PSYCHIATRY

SCHOLARONE™
Manuscripts

Title Page

A Randomized Controlled Trial of Fluoxetine Versus Naltrexone in Compulsive Sexual Behavior Disorder: Presentation of the Study Protocol

Date for submission

03/27/2021

Authors

Family name(s) are typed in **bold**

Josephine **Savard**, MD, PhD[a,b] josephine.savard@umu.se

Katarina **Görts Öberg**, PhD[b,c] katarina.gorts-oberg@sll.se

Cecilia **Dhejne**, MD, PhD[b,c] cecila.dhejne@sll.se

Jussi **Jokinen**, MD, PhD[a, d] jussi.jokinen@ki.se, jussi.jokinen@umu.se

[a] Department of Clinical Sciences/Psychiatry, Umeå University, Umeå, Sweden.

[b] Anova, Karolinska University Hospital, Stockholm, Sweden.

[c] Department of Medicine, Karolinska Institutet, Stockholm, Sweden.

[d] Centre for Psychiatry Research, Department of Clinical Neuroscience, Karolinska Institutet, Stockholm, Sweden.

Corresponding author: Josephine Savard, ANOVA, Karolinska University Hospital, 171 76 Stockholm, Sweden.

Telephone: (+46)8-517 732 00. Fax number: (+46)8-517 718 14. E-mail: josephine.savard@umu.se

Word count: 2922

For peer review only

ABSTRACT

Background: Compulsive Sexual Behavior Disorder is a new disorder in the ICD-11, and is associated with negative consequences in different areas of life. Evidence for pharmacological treatment of compulsive sexual behavior disorder is weak and treatment options are limited. This proposed study will be the largest and the first randomized controlled trial comparing the efficacy and tolerability of two active drugs in Compulsive Sexual Behavior Disorder.

Methods and analysis: Eighty adult participants with Compulsive Sexual Behavior Disorder according to ICD-11 will be randomized to receive either Naltrexone 25-50 mg or Fluoxetine 20-40 mg for eight weeks, followed by six weeks without treatment. The study will be conducted in a sub-specialized outpatient sexual medicine unit at Karolinska University Hospital, Stockholm, Sweden. The study is financed by grants and entirely independent of the manufacturers.

Exclusion criteria include severe psychiatric or psychical illness, changes to concurrent medication, and non-compatible factors contraindicating the use of either drug. The primary outcome measure is the Hypersexual Disorder: Current Assessment Scale (HD: CAS), and tolerability will be assessed by the UKU side effect rating scale, drug accountability, adherence to treatment, and drop-out rate. Participants will complete questionnaires at regular intervals, with the main endpoint for efficacy after 8 weeks (end of treatment) and after 14 weeks (follow-up). Blood chemistry will be repeatedly collected as a safety precaution and for research purposes. The results will be analyzed using an appropriate analysis of variance model or a mixed model, depending on the distribution of HD: CAS and the extent of missing data.

Ethics and dissemination: The Swedish Ethical Review Authority and the Swedish Medical Products Agency have approved the study on 27 May 2020 and 4 June 2020, respectively (Ref. no. 2020-02069 and Ref. no. 5.1-2020-48282). Findings will be published in peer-reviewed journals and presented at relevant conferences.

Registration details: Clinicaltrialsregister.eu, EudraCT: 2019-004255-36 (Cesar study).

For peer review only - http://bmjopen.bmj.com/site/about/guidelines.xhtml

Strengths and limitations of this study

This is the first randomized controlled trial comparing the efficacy of two pharmacological agents in Compulsive Sexual Behavior Disorder- naltrexone and fluoxetine.

The study enables assessment of clinical, psychosocial and biological predictors of treatment response. The lack of placebo-control is a limitation.

The study protocol duration of 14 weeks may be seen as limitation of the study design.

For peer review only

INTRODUCTION

Compulsive Sexual Behavior Disorder (CSBD) is a newly-defined diagnosis in the impulse control disorder section of the 11th edition of the International Classification of Diseases (ICD-11).[1] It entails a persistent pattern of failure to control intense repetitive sexual impulses or urges, resulting in sexual behaviors with negative consequences affecting different areas of life. Adverse consequences associated with CSBD include distress, unwanted pregnancies, sexually transmitted diseases, relationship problems, financial expenses, occupational impairment, and risk of crime. Considering these potential severe consequences and the suggested prevalence of 3-6% of the population,[2] there is an undisputable, urgent need for effective treatments.

Existing drug studies of CSBD have mainly been case reports and small open-label studies on Selective Serotonin Reuptake Inhibitors (SSRIs),[3-4] anti-androgens,[4] or the opioid antagonist naltrexone.[5-8] Only one randomized controlled trial has examined the effect of an SSRI (citalopram 20-60 mg/day for 12 weeks) in 28 gay and bisexual men with non-paraphilic compulsive sexual behavior.[9] There was no significant difference in the main outcome measure Y-BOCS-CSB score between those assigned citalopram and those assigned placebo.

CSBD and Paraphilic Disorders are regarded as separate conditions, however, a high level of sexual pre-occupation is common in both.[10-13] To aid physicians in clinical practice, the World Federation of Societies of Biological Psychiatry (WFSBP) has provided treatment guidelines for paraphilic

For peer review only - http://bmjopen.bmj.com/site/about/guidelines.xhtml

disorders.[14-15] Psychotherapy is proposed as a first step for low-risk individuals. If results are unsatisfactory, the next step includes SSRIs.

The subsequent levels in the guidelines are for individuals with moderate-high risk for sexual violence and include testosterone-lowering agents such as cyproterone acetate (CPA) and gonadotropin-releasing hormone agonists. These agents decrease the frequency and intensity of sexual desire and arousal, however use is associated with high rates of adverse effects. Hormonal treatment is potentially unsuitable for help-seeking individuals with conventional CSBD. In order to investigate appropriate pharmacological alternatives, the proposed 14-week randomized controlled trial will compare the efficacy of two active drugs in 80 adults with CSBD. Additional aims are to examine tolerability and what clinical characteristics and biomarkers could be predictors of response.

Based on the WFSBP treatment guidelines and available research,[9] the SSRI *fluoxetine* will be defined as standard treatment. Alternative treatment will consist of *naltrexone*, an opioid antagonist which prevents reinforcing effects in the mesolimbic reward center and is used in the treatment of alcohol use disorder.[16] Naltrexone was chosen due to similarities between CSBD and other urge-driven disorders, and promising results in CSBD case reports, and in our pilot study of 20 men conducted during 2018-2019.[17] In the latter, all participants completed the eight week study protocol, and no serious adverse events occurred. Although adverse reactions were common, all were considered mild to moderate, and most were transient during the first days-week. Hence, we were able to determine that naltrexone was a well-tolerated, feasible treatment option, and gave indications of symptom relief. However, as the study format prohibited conclusion of efficacy, this study aims to further investigate naltrexone in the treatment of CSBD.

Primary Objective

To compare the effect of naltrexone versus fluoxetine in Compulsive Sexual Behavior Disorder.

Secondary Objectives

To investigate if clinical, psychosocial, or biological factors can predict treatment response.

For peer review only - http://bmjopen.bmj.com/site/about/guidelines.xhtml

To compare if there are any differences in drop-out rate and adherence between the two treatment groups.

To assess side effects and investigate any connection between drop-out rate, reports of side effects and/or efficacy.

To compare if there is a difference in the participants' wish to resume treatment.

To investigate biological markers and clinical parameters in participants with Compulsive Sexual Behavior Disorder.

To investigate correlation between Compulsive Sexual Behavior Disorder and impulsiveness, experience of violence and suicidality.

For peer review only

METHODS AND ANALYSIS

Study setting and study design

The study will be conducted at ANOVA, a subspecialized sexual medicine outpatient clinic at Karolinska University Hospital in Stockholm, Sweden. Eligible participants will be randomized in blocks with a 1:1 allocation to receive either fluoxetine or naltrexone in a superiority parallel group design for eight weeks followed by a six-week follow-up phase. Blinding will not be applicable due to the different appearance of the drugs and dose augmentation in different time intervals due to different pharmacological properties.

Protocol version

Version identifier: 2.0. Number of protocol amendments: 1; 25 June 2020; main changes included adding blood samples and assessments for safety reasons as requested by the Swedish Medical Products Agency.

Eligibility Criteria

Please see Table 1 for inclusion and exclusion criteria.

Table 1. Inclusion and exclusion criteria

For peer review only - http://bmjopen.bmj.com/site/about/guidelines.xhtml

**Inclusion criteria**
Meet criteria for Compulsive Sexual Behavior Disorder according to ICD-11 and fulfill criteria for Hypersexual Disorder as originally proposed for inclusion in DSM-5.
Between 18-65 years.
Understand oral and written Swedish and have internet access.
Willing to participate in all study visits including providing blood and urine samples.
Signed informed consent form.
For fertile women: the use of a safe method of contraception during the entire study protocol.
**Exclusion criteria**
Signs of hepatitis, elevated liver enzymes (> 3 times over reference), or a history of liver failure.
eGFR < 60 ml/min, signs or history of acute kidney failure.
fp-glucose ≥ 7.0 mmol/l.
Known heart disease such as angina pectoris, previous heart failure, or heart attack.
Other serious physical illness including diabetes mellitus, epilepsy, or known ocular hypertension.
Treatment in the past month (≥ 1 dose) with opioids or benzodiazepines.
Treatment in the past month with oral anticoagulants such as warfarin. Intermittent treatment (max. 15 doses per week) with NSAID (e.g. ibuprofen) is tolerated.
Treatment with tamoxifen.
Self-reported use of recreational drugs in the past month or positive drug verification analysis.
Alcohol dependence or risk consumption (> 14 units of alcohol per week for men, > 9 for women) in the past month.
Severe psychiatric disorder requiring immediate treatment such as current psychotic disorder or severe depression.
Bipolar disorder or history of hypomania.
Ongoing treatment with naltrexone or SSRI, or previous hypersensitivity reaction to either.
Change of concurrent medication or dosage in the past three months regarding antidepressants, ADHD medication, mood stabilizers, antipsychotics, cortisone, testosterone, or dopamine precursors. Smaller adjustments may in some cases be acceptable (assessed by study psychiatrist).
Ongoing pharmacological treatment with contraindicated substances (e.g. tamoxifen, metoprolol).
Pregnancy and/or breastfeeding.
Mental condition that could negatively influence either the participant's health or the scientific aspects of the study. High risk for committing sexual offense is included.

For peer review only

For peer review only - http://bmjopen.bmj.com/site/about/guidelines.xhtml

Ongoing psychotherapeutic treatment.
Participation in other studies outside ANOVA.
Abbreviations: ADHD = Attention-Deficit/Hyperactivity Disorder, NSAID = Nonsteroidal anti-inflammatory agents, DSM-5 = Diagnostic and Statistical Manual of Mental Disorders, Fifth Edition.

Recruitment, randomization and allocation

The study will be advertised in media and an initial screening regarding compulsive sexual behavior and inclusion and exclusion criteria (Table 1) will be conducted when potential candidates call the national helpline PrevenTell for individuals with self-identified compulsive sexual behavior and/or paraphilia. Persons likely to meet criteria for CSBD will receive information about the study and be invited to log into a secure web-based platform to leave a preliminary informed consent and fill in questionnaires.

Participants will thereafter attend a baseline visit at the clinic. After obtaining written informed consent (online supplemental file 1), urine samples will be collected and screened for recreational drug use (e.g. amphetamine, benzodiazepines, cannabis, cocaine, and opioid) and blood samples will be collected (e.g. monitoring of testosterone, LH, glucose, electrolytes, and liver enzymes) (Table 2). Specific research samples will also be collected (e.g. DNA extraction for genome-wide methylation analysis (EPIC) and second-generation DNA sequencing).

Table 2. Samples analyzed during the study

**Screening**		
S-FSH	P-ASAT	P-Sodium
S-LH	P-Calcium	
S-SHBG	fP-Glucose	Full blood cell count

For peer review only - http://bmjopen.bmj.com/site/about/guidelines.xhtml

S-Testosterone	P-GT	B-HbA1c
S-TSH	P-HDL Cholesterol	
S-T4	P-Potassium	U-screening for substance abuse [a]
P-Albumin	P-Cholesterol	U-Pregnancy test [b]
P-ALAT	P-Creatinine	
P-ALP	fP-LDL Cholesterol	Research samples [c]
**At 4 weeks**		
P-ALAT	P-GT	Full blood cell count
P-ALP	P-Potassium	
P-ASAT	P-Creatinine	U-Pregnancy test [b]
P-Glucose	P-Sodium	
**At 8 weeks**		
P-ALAT	P-Creatinine	U-Pregnancy test [b]
P-ALP	P-Sodium	P-Fluoxetine or U-Naltrexone [d]
P-ASAT	Full blood cell count	
P-GT	B-HbA1c	Research samples [c]
P-Potassium		

Notes: [a] Amphetamine, benzodiazepines, buprenorphine, cannabis, cocaine, fentanyl, methadone, oxycodone, tramadol, and other opioids.
[b] Assigned female at birth only
[c] e.g. DNA extraction for genome-wide methylation analysis (EPIC) and second-generation DNA sequencing, oxytocin pre- and post-treatment.
[d] Depending on randomization

A psychiatrist will obtain a medical and psychiatric history, perform a physical examination including blood pressure and heart and pulmonary auscultation, as well as perform interviews with the Mini International Neuropsychiatric Interview (MINI)[18] and Columbia Suicide Severity Rating Scale (C-SSRS).[19] The study psychologist will focus on sexual behaviors by conducting a structured interview

For peer review only - http://bmjopen.bmj.com/site/about/guidelines.xhtml

addressing the ICD-11 criteria for CSBD and the Diagnostic and Statistical Manual of Mental Disorders, Fifth Edition (DSM-5) criteria for Hypersexual Disorder as originally proposed for inclusion[20] and paraphilia(s). The separate psychiatrist and psychologist interviews aim to determine eligibility criteria in an unbiased manner. If unmatched opinions, the participant will not be included.

The research nurse will open envelopes for randomization and supply enrolled participants with either naltrexone or fluoxetine accordingly. The allocation sequence is created by an independent investigator at the Karolinska Trial Alliance, a research center that supports clinical trials. Participants will start treatment if results on blood chemistry are adequate.

The study drugs will be provided by the Karolinska University Hospital's pharmacy.

Naltrexone (AOP Orphan Pharmaceuticals): Initial dose 25 mg per day, and if tolerated will be augmented to 50 mg per day after 3-5 days.

Fluoxetine (Orion Pharma): Initial dose 20 mg per day, and if unsatisfactory symptom reduction and tolerated will be augmented to 40 mg per day after four weeks.

In Sweden, The Dental and Pharmaceutical Benefits Agency, a central government agency determines what pharmaceutical product shall be subsidized by the state. The agency also recommends manufacturers for specific drugs based on e.g. prize and availability. The manufacturers for naltrexone and fluoxetine in this study were chosen as they were the agency's recommended product when the application was prepared for submission.

Intervention

Figure 1 illustrates study procedures. Participants will assess their symptoms weekly by filling in questionnaires online e.g. if they have noticed any change in frequency or intensity of sexual urges or behaviors, how they perceive the treatment and if they experience adverse reactions. Every second week, participants will complete the main outcome measure Hypersexual Disorder: Current Assessment Scale (HD: CAS).[21] After four weeks, new blood samples will be collected, and participants will have a telephone consultation with the study psychiatrist to assess tolerability and psychiatric well-being. Fertile women will be required to conduct a pregnancy test before starting the study and use contraception during the study period.

At end of treatment, participants will have a consultation with the study psychiatrist to assess current

For peer review only - http://bmjopen.bmj.com/site/about/guidelines.xhtml

symptoms of CSBD, psychiatric distress, and tolerability. Blood samples will be collected for chemical analysis as well as monitoring drug tablet return and the metabolites of naltrexone respective fluoxetine.

Six weeks after the end of treatment, the participants will fill in questionnaires and have a final consultation with the study psychiatrist. The visits at the clinic and the drugs will be free of charge.

Outcome measures

The main outcome is symptom relief as assessed with HD: CAS, which measures symptom severity during the previous two weeks according to the suggested conceptualization of Hypersexual Disorder to the DSM-5.[20] Corresponding scales for the ICD-11 diagnosis have not yet been developed. We chose a scale that is sensitive to changes and participants will be asked to fill it in at baseline, every second week during the treatment phase, and at end of study. However, as the scale is not validated, two additional measures will be used: the Hypersexual Behavior Inventory (HBI)[22] and the Sexual Compulsivity Scale (SCS).[23] Using unpublished data from our research center, the correlations (Pearson's) between HD: CAS and HBI were positive at baseline and post-treatment.

We will also analyze factors (clinical, psychosocial, or biological) that may be predictors of response, and whether there is any difference in participants' readiness to resume pharmacological treatment at end of study.

Furthermore, we will also evaluate tolerability using the UKU side effect rating scale,[24] drug accountability, adherence to treatment, and drop-out rate.

Finally, to aid in understanding CSBD, we will assess for impulsivity, suicidality, and childhood adversities, and collect biological markers (e.g. DNA and hormones) to evaluate their potential association with CSBD. A summary of assessments and biological markers is presented in Table 2, Table 3, and supplemental Table (online supplemental file 2). Furthermore, an extension of the study is planned to include neuroimaging data collection.

Table 3. Study assessments

Objective	Assessments	Measure type	Time point
Assess changes in compulsive sexual behaviors from screening to week 8 and follow up	Hypersexual Disorder: Current Assessment Scale (HD: CAS)	Outcome	Baseline, week 2, 4, 6, 8 and end of study

For peer review only - http://bmjopen.bmj.com/site/about/guidelines.xhtml

Assess compulsive sexual behaviors	The Hypersexual Disorder Screening Inventory (HDSI)	Eligibility	Baseline, and end of study
Assess changes in compulsive sexual behaviors from screening to week 8 and follow up	The Hypersexual Behavior Inventory (HBI)	Outcome	Baseline, week 1-4, week 6, week 8, and end of study
Assess changes in compulsive sexual behaviors from screening to week 8 and follow up	Sexual Compulsivity Scale (SCS)	Outcome	Baseline, week 1-4, week 6, week 8, and end of study
Assess sexual interests	Self-assessment of sexual interests (SSI)	Eligibility	Baseline
Assess sexual dysfunction	International Index of Erectile Function (IIEF)[a]	Eligibility	Baseline
Assess psychiatric co-morbidity	Mini International Neuropsychiatric Interview (MINI)	Eligibility	Baseline
Assess changes in depression severity from screening to week 8 and follow up	Montgomery Åsberg Depression Rating Scale – Self-rating (MADRS-S)	Outcome Safety	Baseline, week 8, and end of study
Assess change of symptoms of depression and anxiety from screening to week 8 and follow up	Hospital Anxiety and Depression Scale (HAD)	Outcome	Baseline, week 8, and end of study
Assess changes in alcohol use from screening to week 8 and follow up	Alcohol Use Disorders Identification Test (AUDIT)	Outcome	Baseline, week 8, and end of study
Assess use of drugs	Drug Use Disorders Identification Test (DUDIT)	Eligibility	Baseline
Assess pathological gambling	Gambling Disorder Identification Test (G-DIT)	Eligibility	Baseline
Assess impulsivity	Barratt Impulsiveness Scale (BIS-11)	Eligibility	Baseline
Assess symptoms of Attention-Deficit/ Hyperactivity Disorder	Adult ADHD Self-Report Scale (ASRS)	Eligibility	Baseline
Assess history of violence	Karolinska Interpersonal Violence Scale (KIVS)	Eligibility	Baseline

For peer review only

Assess history of childhood trauma	The Childhood Trauma Questionnaire – Short Form (CTQ-SF)	Eligibility	Baseline
Assess suicide ideation and suicidal behavior throughout the study	Columbia Suicide Severity Rating Scale (C-SSRS)	Safety	Baseline, week 1, 4, 8, and end of study
Assess adverse reactions in week 8	UKU side effect rating scale (UKU)	Tolerability	Week 8
Question of wanting to resume pharmacological treatment, week 14	Yes/No/Don't know option	Outcome	End of study
Notes: [a] assigned men at birth only.			

Adherence and Concomitant Care

Participants can leave the study at any time without giving a reason, however any material already obtained will be analyzed. Participants who miss ≥ 3 doses in a row will be considered as having ceased treatment. Other reasons for discontinuation of treatment include severe adverse reactions, severe psychiatric condition where the safety of the participant or others cannot be guaranteed, initiation of treatment with non-compatible drugs or use of recreational drugs, pregnancy, or start of psychotherapy.

Sample Size and Statistical Methods

Since there is no gold standard for the measurement of pharmacological treatment outcomes in CSBD, the sample size calculation is based on HD: CAS from a study of Cognitive Behavioral Therapy from our clinic[25] (using expanded data available post-publication, n = 76). The difference in HD: CAS pre-post treatment was 4.065 with a standard deviation of 4.74. A statistical power of 80 %, an error probability of 0.05, and a difference between the groups of 3.3 (meaning an effect size of 0.7) would render a sample size of 34 participants in each group. To adjust for potential dropouts, we aim to include 80 participants. The results will be analyzed using an appropriate analysis of variance model or a mixed model, depending on the distribution of HD: CAS and the extent of missing data. Participants who discontinue treatment will be included in an intention-to-treat analysis. Per-protocol analysis will also be used. To increase the study's credibility, the extent and nature of missing data will be reported for each treatment group, as well as any predictors of missing data (e.g. young age).

Monitoring

As required, an independent trial investigator from the Karolinska Trial Alliance will regularly control and quality assure the study procedures. There will be no interim analysis.

For peer review only - http://bmjopen.bmj.com/site/about/guidelines.xhtml

Patient and public involvement

Patients and the public will not be involved in the design of the study other than the input from the participants and outcomes from the pilot study.[17]

Ethics and dissemination

The study procedures will be carried out in accordance with the Declaration of Helsinki and European Good Clinical Practice Guidelines. The Swedish Ethical Review Authority and the Swedish Medical Products Agency have approved the study (Ref. no. 2020-02069 and Ref. no. 5.1-2020-48282). The registration of the trial (Eudra-CT no. 2019-004255-36) is accessible at https://www.clinicaltrialsregister.eu. Study enrollment started in October 2020.

Eligible participants will be aged ≥ 18 years and understand both oral and written Swedish. Paraphilic interests are not an exclusion criterion, whereas severe risk for sexual violence (as reported in the interviews and questionnaires, e.g. participants who report that they actively interact with children for sexual purposes) is.

The study psychiatrists and psychologists are experienced in evaluating and treating patients with CSBD and Paraphilic Disorders, and the investigators will together decide whether the individual may be a potential study candidate.

The risks for the participants including safety aspects of the drugs have been carefully weighed against the potential benefits of conducting the study, and we believe the latter outweighs the former. The participants' health and safety will be assessed and prioritized, which may entail discontinuation of treatment. All undesirable medical events that come to our attention will be graded (mild if the symptoms are transient, moderate if temporary medical treatment for relief is needed, and severe if hospital care or prolonged medical treatment is needed) and followed until the event is resolved. Serious adverse events and suspected unexpected serious adverse reactions are handled and reported as required by the Swedish Medical Products Agency. The same insurance regulation as in ordinary medical care apply.

Research data from the web-based platform will be stored securely in servers with restricted access. Archived paper material will be stored in a locked cabinet, in accordance with current legislation and regulations. Research samples and data will be destroyed 10 years after declaring end of trial to the Swedish Medical Products Agency.

Authorship eligibility will be assured in accordance with The International Committee of Medical Journal Editors (ICMJE) guidelines The results of the study will be presented at scientific conferences and in peer-review articles. Once the trial has been published, participants will be informed about the study results on a group level and results suitable for a non-specialist audience will be accessible on our website.

For peer review only - http://bmjopen.bmj.com/site/about/guidelines.xhtml

DISCUSSION

As presented in the introduction, there is currently weak evidence for the pharmacological treatment of CSBD (for review, see Grubbs et al.[26]) and hence a clear need for randomized controlled trials. However, some aspects of this study design and concept need to be discussed:

Feasibility of enrollment

Participants might be unwilling to participate in a drug trial, hence it may be difficult to complete recruitment within the designated period of three years, leading to a sample too small for rending power in the analyses. In addition, the Covid-19 pandemic might complicate participants' travel to our clinic or even our capacity to conduct the study due to restrictions in the provision of healthcare (e.g. limited to emergency care only). An overrepresentation of participants living in the Stockholm area is to be expected.

Eligibility

We try to minimize the risk for recruiting a homogeneous group of persons with CSBD by inviting individuals with a broad range of severity of symptoms, and the use of randomization with allocation concealment to minimize the risk for selection bias between the groups. Nevertheless, as with most studies, there is a risk for an overrepresentation of those with a better prognosis.
The exclusion criteria are more stringent than the inclusion criteria, partly due to the safety profiles of the drugs – a situation that regardless of study protocol would influence their use in a regular clinical setting. However, we also exclude participants with ongoing recreational drug use to minimize the risk of confounders. We also exclude participants currently being treated with interacting antidepressants and those with a severe psychiatric disorder requiring immediate treatment, as these patients are too sick to follow our study protocol. Most patients seeking treatment at ANOVA for CSBD do not fall into these categories (particularly not the latter), but this exclusion criterion will undoubtedly lead to the inclusion of healthier individuals in the study. Although we aim to have a representative sample, a certain impact on the external validity is to be expected.

Outcome ascertainment

As noted, our main outcome variable HD: CAS is not validated. However, it has a predefined timespan of symptom relief and has been used in previous studies at our research center. When comparing results of HD: CAS with the validated HBI, the latter reaches a floor-effect post treatment, whereas HD: CAS seems to better distinguish effects. Our overall judgement is thus that the advantages of the HD: CAS outweigh the disadvantages.
The HBI has an item addressing craving "My sexual cravings and desires feel stronger than my self-

For peer review only - http://bmjopen.bmj.com/site/about/guidelines.xhtml

discipline". Nevertheless, we are aware that using this single item to assess craving may be a limitation.

Another limitation is that this study will not be blinded, and we will not be able to control for the anticipation effect in either group. Nonetheless, group comparisons using regression models with incorporated baseline data will still be of major value and results will be of importance to guide clinical praxis and for future meta-analyses. Finally, the study protocol duration of only 14 weeks is a major limitation, but the length was chosen for feasibility reasons. Even so, if adherence to naltrexone will be low, future studies could consider using extended-release injectable naltrexone. Fluoxetine will presumably reach steady state between week four and eight, and with this aspect in mind the comparisons between the groups after reaching steady state will be the most informative as well as being deemed sufficient to provide important clinical knowledge.

Acknowledgements

The authors wish to thank Tania McConaghy for language revision.

Contributors

JS, KGÖ, CD and JJ conceived and designed the study. JS and JJ contributed to the statistical plan. JJ is grant holder. JS drafted the manuscript, and all author contributed substantially to refinement of the manuscript. All authors approved the final version.

Funding

This work was supported by the Swedish Research Council grant number 2020-01183 and regional agreements between ANOVA, Region Stockholm, and Region Västerbotten (ALF Region Västerbotten grant number RV-929554). The pharmaceutical companies are not providing funding or being involved in any other way.

Competing interests

Jussi Jokinen has participated in Advisory Board of Janssen concerning esketamine for MDD with current suicidal ideation. The other authors report no conflicts of interest.

Data sharing statement

Individual participant will be available under certain circumstances to researches who provide a methodically sound proposal.

The study protocol and individual participants' data that underlie the results of the published article, after deidentification (text, tables, figures, and appendices) will be available to achieve aims in the approved proposal. Data will become available immediately following publication and ending nine years following article publication.

Proposals should be directed to Josephine.savard@umu.se. To gain access, data requestors will need to sign a data access agreement.

For peer review only - http://bmjopen.bmj.com/site/about/guidelines.xhtml

Figure 1. Flowchart.

Note: * If unsatisfactory symptom reduction and tolerated.

REFERENCES

1. World Health Organization. The 11th revision of the International Classification of Diseases (ICD-11). Available from: https://icd.who.int/browse11/l-m/en#/http%3a%2f%2fid.who.int%2ficd%2fentity%2f274880002 accessed February 9, 2020.
2. Kuzma JM, Black DW. Epidemiology, prevalence, and natural history of compulsive sexual behavior. *Psychiatr Clin North Am* 2008;31(4):603-11. doi: 10.1016/j.psc.2008.06.005
3. Gola M, Potenza MN. Paroxetine Treatment of Problematic Pornography Use: A Case Series. *Journal of Behavioral Addictions* 2016;5(3):529-32. doi: 10.1556/2006.5.2016.046
4. Winder B, Lievesley R, Elliott H, et al. Evaluation of the use of pharmacological treatment with prisoners experiencing high levels of hypersexual disorder. *J Forens Psychiatry Psychol* 2018;29(1):53-71. doi: 10.1080/14789949.2017.1337801
5. Raymond NC, Grant JE, Kim SW, et al. Treatment of compulsive sexual behaviour with naltrexone and serotonin reuptake inhibitors: two case studies. *Int Clin Psychopharmacol* 2002;17(4):201-5. doi: 10.1097/00004850-200207000-00008
6. Ryback RS. Naltrexone in the treatment of adolescent sexual offenders. *J Clin Psychiatry* 2004;65(7):982-86. doi: 10.4088/JCP.v65n0715
7. Raymond NC, Grant JE, Coleman E. Augmentation with naltrexone to treat compulsive sexual behavior: A case series. *Ann Clin Psychiatry* 2010;22(1):56-62.
8. Bostwick JM, Bucci JA. Internet sex addiction treated with naltrexone. *Mayo Clin Proc* 2008;83(2):226-30. doi: 10.4065/83.2.226
9. Wainberg ML, Muench F, Morgenstern J, et al. A double-blind study of citalopram versus placebo in the treatment of compulsive sexual behaviors in gay and bisexual men. *J Clin Psychiatry* 2006;67(12):1968-73. doi: 10.4088/JCP.v67n1218
10. Kafka MP, Hennen J. The paraphilia-related disorders: an empirical investigation of nonparaphilic hypersexuality disorders in outpatient males. *J Sex Marital Ther* 1999;25(4):305-19. doi: 10.1080/00926239908404008
11. Sutton KS, Stratton N, Pytyck J, et al. Patient Characteristics by Type of Hypersexuality Referral: A Quantitative Chart Review of 115 Consecutive Male Cases. *J Sex Marital Ther* 2015;41(6):563-80. doi: 10.1080/0092623x.2014.935539
12. Kafka MP, Hennen J. Hypersexual desire in males: are males with paraphilias different from males with paraphilia-related disorders? *Sex Abuse* 2003;15(4):307-21. doi: 10.1177/107906320301500407
13. Engel J, Veit M, Sinke C, et al. Same Same but Different: A Clinical Characterization of Men with Hypersexual Disorder in the Sex@Brain Study. *J Clin Med* 2019;8(2) doi: 10.3390/jcm8020157
14. Thibaut F, De La Barra F, Gordon H, et al. The World Federation of Societies of Biological Psychiatry (WFSBP) guidelines for the biological treatment of paraphilias. *World J Biol Psychiatry* 2010;11(4):604-55. doi: 10.3109/15622971003671628
15. Thibaut F, Cosyns P, Fedoroff JP, et al. The World Federation of Societies of Biological Psychiatry (WFSBP) 2020 guidelines for the pharmacological treatment of paraphilic disorders. *The World Journal of Biological Psychiatry* 2020;21(6):412-90. doi: 10.1080/15622975.2020.1744723
16. Franck J, Jayaram-Lindstrom N. Pharmacotherapy for alcohol dependence: status of current treatments. *Curr Opin Neurobiol* 2013;23(4):692-9. doi: 10.1016/j.conb.2013.05.005
17. Savard J, Öberg KG, Chatzittofis A, et al. Naltrexone in Compulsive Sexual Behavior Disorder: A Feasibility Study of Twenty Men. *J Sex Med* 2020 doi: 10.1016/j.jsxm.2020.04.318

For peer review only - http://bmjopen.bmj.com/site/about/guidelines.xhtml

18. Sheehan DV, Lecrubier Y, Sheehan KH, et al. The Mini-International Neuropsychiatric Interview (M.I.N.I.): the development and validation of a structured diagnostic psychiatric interview for DSM-IV and ICD-10. *J Clin Psychiatry* 1998;59 Suppl 20:22-33;quiz 34-57.
19. Posner K, Brown GK, Stanley B, et al. The Columbia–Suicide Severity Rating Scale: Initial Validity and Internal Consistency Findings From Three Multisite Studies With Adolescents and Adults. *Am J Psychiatry* 2011;168(12):1266-77. doi: 10.1176/appi.ajp.2011.10111704
20. Kafka MP. Hypersexual disorder: a proposed diagnosis for DSM-V. *Arch Sex Behav* 2010;39(2):377-400. doi: 10.1007/s10508-009-9574-7 [published Online First: 2009/11/26]
21. American Psychiatric Association. Hypersexual Disorder: current assessment scale. American Psychatric Association's DSM-V workgroup on Sexual and Gender Identity Disorders 2010. Available from: http://www.dsm5.org/ProposedRevisions/Pages/proposedrevision.aspx?rid=415 accessed March 2010.
22. Reid RC, Garos S, Carpenter BN. Reliability, Validity, and Psychometric Development of the Hypersexual Behavior Inventory in an Outpatient Sample of Men. *Sexual Addiction & Compulsivity* 2011;18(1):30-51. doi: 10.1080/10720162.2011.555709
23. Kalichman SC, Rompa D. Sexual sensation seeking and Sexual Compulsivity Scales: reliability, validity, and predicting HIV risk behavior. *J Pers Assess* 1995;65(3):586-601. doi: 10.1207/s15327752jpa6503_16
24. Lindstrom E, Lewander T, Malm U, et al. Patient-rated versus clinician-rated side effects of drug treatment in schizophrenia. Clinical validation of a self-rating version of the UKU Side Effect Rating Scale (UKU-SERS-Pat). *Nord J Psychiatry* 2001;55 Suppl 44:5-69. doi: 10.1080/080394801317084428
25. Hallberg J, Kaldo V, Arver S, et al. Internet-Administered Cognitive Behavioral Therapy for Hypersexual Disorder, With or Without Paraphilia(s) or Paraphilic Disorder(s) in Men: A Pilot Study. *J Sex Med* 2020;17(10):2039-54. doi: 10.1016/j.jsxm.2020.07.018
26. Grubbs JB, Hoagland KC, Lee BN, et al. Sexual addiction 25 years on: A systematic and methodological review of empirical literature and an agenda for future research. *Clin Psychol Rev* 2020;82:101925. doi: https://doi.org/10.1016/j.cpr.2020.101925

For peer review only

For peer review only - http://bmjopen.bmj.com/site/about/guidelines.xhtml

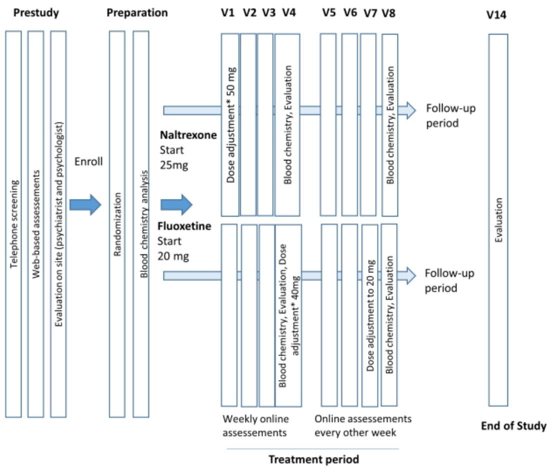

Flowchart.

338x190mm (300 x 300 DPI)

INFORMED CONSENT FORM

Pharmacological treatement of Compulsive Sexual Behavior

- a comparison of the effectiveness of naltrexon versus fluoxetin: the CeSar-study

Background and aim

Here at ANOVA clinic we treat persons who experience a troublesome or dangerous sexuality. There is a lack of knowledge regarding treatment of intrusive sexual thoughts, impulses and behaviors in individuals with compulsive sexuality, sometimes called “sexual addiction”. This study is evaluating a new drug for this purpose, naltrexone. Naltrexone is currently used in the treatment of alcohol dependence and has been reported as a drug that can also reduce both urges and behaviors linked to compulsive sexual behavior. To compare the new treatment, half of the participants will instead receive fluoxetine which is used for the treatment of compulsive sexuality nationally and internationally.
The overall purpose of this study is to improve the treatment options and the quality of life for the individual.

Request for participation

You have contacted or been referred to ANOVA and are therefore asked to participate. Participation is voluntary.

How is the study conducted?

If you are interested in participating in the study and provide an informed consent in the web platform, you will be asked to complete an online survey for approximately 60-90 minutes with questions on your sexuality and your general well-being. The online survey is the first step in the study and aims to provide increased knowledge about sexuality that leads to negative consequences.
After you have filled in the online survey, you will be contacted by mail or telephone and offered an appointment at the clinic ANOVA. You will meet a physician and psychologist for two interviews. They will evaluate if you meet the requirements to be enrolled in the study and you will have the opportunity to ask questions. If you want to proceed with the study, you will be requested to sign an informed consent for participation in the drug treatment. You will also be asked to participate in a computerized test of impulsivity and to submit a routine blood test to map sex hormones and liver-, thyroid- and kidney function and metabolic status (= 5 tubes, approximately 20ml) before treatment. We also ask for a urine sample to have objective measures to exclude a recreational drug use. This urine sample is discarded if it is negative or sent to a laboratory for further analysis if positive as done with routine sample in healthcare. If you are a woman of childbearing age, you will have to submit a sample (urine or blood sample, a 3.5ml tube) to rule out pregnancy and are encouraged to use a safe contraception method during the study.

For peer review only

We also ask if you are willing to provide research blood samples (max. 100ml) with the aim to examine neurobiological markers of compulsive sexuality. These samples include e.g. DNA extraction, see further under the heading Biobank below.

Estimated time required for the visits is about 3 hours.

If you are offered treatment within the framework of the study, you will receive treatment for 8 weeks with either naltrexone or fluoxetine. The selection is made through randomization (i.e. assignment by chance). You will know which of the preparations you have been randomized to, but will not have the opportunity to choose drug.

You will be contacted by phone after 3-5 days and after 4 weeks to inquire about mood and possibly adjust the dose. At 4 weeks, you will be asked to provide new blood samples (3 tubes, approx. 12 ml).
You will be asked to report any side effects and if you notice any changes since the start of medication in the web-based platform. In addition, you will be asked to fill questionnaires every week that assess you mood and your sexual problems. Filling in questionnaires will take about 10-20 minutes per week.
You will be contacted and offered a psychologist or physicians' appointment if we become concerned about your mental or physical state.

Visit 2 at ANOVA: After finishing treatment, you will have a follow-up visit with a physician. You will also be asked to provide urine / blood to see the concentration of the drug in the body, routine blood tests (4 tubes, approx. 16 ml) and research blood samples (2 tubes, approx. 8 ml) to examine whether the treatment has i.e. affected hormone levels.

Visit 3 at ANOVA: Six weeks after the end of treatment, you have a final appointment with a physician who follows up on your mood and your sexuality.

Visits 2 and 3 take about 30-60 minutes each.

If you need care after the trail, you will be guided to the type of care that best suits you. Regardless of whether you are offered treatment or not, your answers in the online survey will be an important part of the study.

Biobank

Routine blood samples will be handled and destroyed according to the hospital's guidelines as in normal sampling in healthcare. The samples that are intended to be stored (5 tubes, max 30 ml) for e.g. DNA analysis and measurement of the hormone oxytocin will be stored in a biobank. The responsible biobank is Stockholms Medicinska Biobank (reg. No. 914) in accordance with the Act on Biobanks in Health Care (2002: 297), which regulates the manner

For peer review only

in which samples may be saved and used, and it also regulates the quality and safety of biobanks.
The samples will be stored coded, which means that the samples cannot be directly traced to you as a person. The samples and the associated identification list (code key) will be kept separate from each other, and protected from access by unauthorized persons.

Coded samples may be sent for analysis both within Sweden and the EU / USA.

Future projects: The blood samples will be stored pending analysis for up to 10 years, after which they will be destroyed. The samples may also be relevant for not yet unplanned research projects if you approve storage for this. A new ethical application will be made in such case, and you may be contacted again with a request for your consent. A separate request regarding future projects is provided in the enclosed consent form.

If you regret that you gave permission for your sample to be stored, you have the right to have the samples destroyed or deidentified by contacting test leader Josephine Savard, please find contact information below.

Are there any risks associated with the study?

Answering questions on private topics such as sexuality and mental health might feel uncomfortable, but these questions need to be asked so we can help you.
As for the medicine, you can experience side effects that are usually transient such as nausea, headache, vomiting and weakness. Serious side effects are uncommon with both drugs. For safety reasons, you will have to provide blood samples before and after the treatment to map, among other things, liver function. Should your samples be abnormal, we will process it according to medical practice. If you are a woman of reproductive age, you need to use safe contraception throughout the study period and submit a negative pregnancy test to be included in the study.

While blood sampling, you may experience temporary discomfort, dizziness and you may get a bruise.

During the course of the study, you could use drugs temporary such as painkillers (e.g. paracetamol) or short-term anti-anxiety medication (e.g. Atarax). Other treatment should be avoided and may lead to discontinuation from the study. This specially applies to drugs with addictive features. Ongoing drug treatment is usually suitable to continue with, but dose changes should be avoided. If in doubt, discuss with your treating physician at ANOVA.

Warning # 1! Should you develop symptoms such as jaundice / hepatitis (inflammation of the liver), you must immediately contact an emergency department or call 112. Symptoms of jaundice are feelings of illness, yellow staining of the skin and whites of the eyes, abdominal pain, light stools and yellow urine.

For peer review only

Warning # 2! Naltrexone counteracts the effect of opioids found in e.g. painkillers (Citodon, OxyContin /OxyNorm, Dolcontin, morphine, etc.) and in cough medicines (Cocillana-Etyfin). There are also opioids in loperamide (Dimor, Loperamide, Imodium) used to treat diarrhea. Should an emergency situation arise where you need treatment with opioids, you should immediately stop taking the study drug and inform the responsible physician about the simultaneous participation in this study.

Warning # 3! Should you during the course of treatment deteriorate mentally with e.g. suicidal ideation, you must contact the study physiatrist or psychiatric emergency service at your place of residence. You will receive contact information at the start of study.

We recommend that you always carry the plastic card you are assigned at start of the study. The card has contact information to the study supervisor so that the treatment unit can obtain information about the study

Are there any benefits to participating in the study?

There is a possibility that you will feel relieved to have shared your thoughts and feelings with a professional and that the drug takes the edge off the desire to commit sexual acts. It can also be an advantage to have contacted our clinic to enroll as a "regular patient" after the study period is over.

Data management and confidentiality

Information about you will be registered within the framework of the study:
- Self-assessments are handled in a web-based platform that is encrypted and protected with double authentication requirements on a secure server at Karolinska Institutet.
- Other data such as interview forms and biobank samples will be handled pseudonymised, i.e. linked to you through a study code. The code key will be kept at ANOVA and protected from access by unauthorized persons.
Your answers and results will be stored in locked folders, secure digital files, encrypted databases and servers in accordance with the General Data Protection Regulation (GDPR 2016/619). Your information will not be used commercially and will be stored for a maximum of 10 years after the end of the study. All information will be handled with total confidentiality. It will not be possible to link any information to you as an individual when the results are reported.

As in ANOVA's regular care, computer-based patient records will be generated. These will not be visible outside ANOVA's record domain.

In order to verify that the collected data is handled correct, and that the procedures are performed in accordance with applicable laws and regulations, the implementation of the research study will be reviewed by an independent trial investigator. Investigators who review

For peer review only

the research study may be given access to decoded data and patient records together with your responsible physician and under confidentiality.

Personal data is protected and processed in accordance with the General Data Protection Regulation (GDPR 2016/679) and the Public Access to Information and Secrecy Act (2009: 400). No unauthorized person will be allowed to access the information. According to the General Data Protection Regulation, you have the right to obtain the information that is handled of you in the study free of charge and, if necessary, have any errors corrected.

If you want to take part of the information, you can contact test leader Dr Josephine Savard for more information, contact information can be found on the last page under the section Responsible. Responsible for the management of personal data is Karolinska University Hospital's Data Protection Office. The Data Protection Officer can be reached at: Karolinska University Hospital, 171 76 Solna, tel. 08-517 700 00 (switchboard operator), e-mail: dataskyddsombud.karolinska@regionstockholm.se. If you are dissatisfied with how your personal data is processed, you have the right to submit a complaint to the Swedish Authority for Privacy Protection, which is the supervisory authority.

How do I get information about the results of the study?

The results will be published in scientific journals. Only statistical variables will be presented, and it will not be possible to identify individual participants. If you want to take part of the scientific reports, you are welcome to contact ANOVA where this study will be carried out. Information on clinical trials can be found at: www.clinicaltrials.gov

Insurance and compensation

The regular patient insurance applies. The drugs and the visits will be free of charge.

Participation is voluntary

Participation in the study is voluntary and you can withdraw your participation at any time without stating why and without it affecting your future care at ANOVA. If you want to discontinue your participation, contact the study supervisor or the test leader (contact information below). After you withdraw your participation, no new information about you will be saved. Already collected data will be preserved.
Samples: If you regret that you gave permission for us to store your samples, you have the right to have the samples discarded (this means that the samples will be destroyed or deidentified).

Responsible for the trial

Responsible for the implementation of the study is ANOVA, Karolinska University Hospital. The principal researcher is Professor Jussi Jokinen (jussi.jokinen@ki.se)

For peer review only

For questions, please contact test leader Josephine Savard, research nurse Susanne Jarlvik Alm or research assistant Pia Jaensson

Dr. Josephine Savard (Testleader)
ANOVA, Karolinska Universitetssjukhuset, 171 76 Stockholm.
E-post: josephine.savard@regionstockholm.se, Tel +46 (0)72-5823241

Susanne Jarlvik Alm
ANOVA, Karolinska Universitetssjukhuset, 171 76 Stockholm.
E-post: Susanne.jarlvik-alm@regionstockholm.se, Tel +46 (0) 8-51772935

Pia Jaensson
ANOVA, Karolinska Universitetssjukhuset, 171 76 Stockholm.
E-post: pia.jaensson@regionstockholm.se, Tel +46 (0) 8-51773832

For peer review only

Consent form: Drug treatment for compulsive sexuality, CeSar study.

• I have read the written information regarding the research study and I have had the opportunity to ask questions and have them answered.
• I provide my consent to participate in the study and know that my participation is completely voluntary. I am aware that blood and urine samples need to be provided based on clinical indication. I regulate how the samples are saved (see below).
• I am aware that I can withdraw my consent and terminate my participation at any time and without explanation, as well as having collected samples discarded. The samples will then be destroyed or deidentified.
• I allow my personal information to be registered according to the information I have received and that collected data about me is stored and handled electronically by study supervisors. I allow unidentified data to be analyzed in future, unplanned studies after approval by the Ethics Review Authority.
• I allow the study supervisor or a study monitor to receive patient record information that is relevant to the current research study and that the Swedish Medical Products Agency and the corresponding supervisory authority within and outside the EU receive patient record information during any supervision.

Signature Name in block letters Date

----------------------------- --------------------------------- --------------------

Consent, research on blood samples within the framework of the study: I give my consent that the samples I submit will be saved in the biobank and that the samples are used for research in accordance with what is described in the patient consent information.

Yes □ No □

Consent, preservation of samples for future research studies: Your samples may be valuable for unplanned research projects (as described in this information) if you approve storage for this. In these cases, a new ethical review will take place and you may be contacted again with a request. Do you consent to that samples that remain after the completion of the Cesar study are stored for future research and may be analyzed within Sweden, the EU and the USA?

Yes □ No □
Signature Name in block letters Date

------------------------------- --------------------------------- --------------------

To be completed by the investigator: I confirm that I have provided both oral and written information about the described trial and that a copy of the patient information sheet has been handed out to the patient.

Signature Name in block letters Date

------------------------------- ---------------------------------- --------------------

For peer review only

Supplementary Table
Outcome Measures with Psychometric Properties

Name	Psychometric properties
The Mini International Neuropsychiatric Interview (MINI)	Validated<br>(Sheehan et al., 1998).
Hypersexual Disorder: Current Assessment Scale (HD:CAS)	Not validated<br>Cronbach's α = 0.76<br>(Savard., 2021)
The Hypersexual Behavior Inventory (HBI)	Validated<br>test-retest reliability r = 0.91; Cronbach's α = 0.96<br>(Reid et al., 2011)
Self-assessment of Sexual Interest (SSI)	Not validated<br>(Långström, unpublished)
The Hypersexual Disorder Screening Inventory (HDSI)	Validated in Swedish<br>inter-rater reliability r = 0.51, Cronbach's α = 0.80-0.81<br>(Öberg et al., 2017)
International Index of Erectile Function (IIEF)	Validated<br>test–retest reliability r = 0.64–0.84; Cronbach's α = 0.73–0.99) across studies.<br>(Rosen et al., 1997; 2002)
Sexual Compulsivity Scale (SCS)	Cronbach's α = 0.89-0.92<br>(Kalichman & Rompa, 1995)
The Alcohol Use Disorders Identification Test (AUDIT)	Validated in Swedish<br>test-retest reliability r = 0.97; Cronbach's α 0.82<br>(Bergman & Kallmen, 2002)
The Drug Use Disorders Identification Test (DUDIT)	Validated in Swedish<br>Cronbach's α = 0.80; sensitivity (ranging from 0.85- 1.00) and specificity (ranging from 0.75 to 0.92)<br>(Berman et al., 2005; Hildebrand 2015)
The Gambling Disorder Identification Test (G-DIT)	Test-retest reliability<br>intraclass correlation coefficient = 0.93<br>Cronbach's α = 0.94<br>(Molander et. al., 2021)
The Childhood Trauma Questionnaire – Short Form (CTQ-SF)	Validated in Swedish<br>The inter-correlations between CTQ total scale and subscales vary between r = 0.15-0.89; Cronbach's α = 0.92 on the total score, subscales α = 0.65-0.86<br>(Bernstein & Fink, 1998; Gerdner & Allgulander 2009)

For peer review only

Karolinska Interpersonal Violence Scale (KIVS)	Validated in Swedish<br>inter-rater reliability for the subscales *r* = 0.91-0.95<br>(Jokinen et al., 2010)
Adult ADHD Self-Report Scale (ASRS)	Validated<br>test-retest reliability *r* = 0.58-0.77<br>(Kessler et al., 2007)
The Barratt Impulsiveness Scale (BIS)	Validated<br>test-retest reliability Spearman's rho = 0.83<br>Cronbach's *α* = 0.83<br>(Stanford et al., 2009)
Montgomery Åsberg Depression Rating Scale – Self-rating (MADRS-S)	Validated in Swedish<br>inter-rater reliability *r* = 0.87; Cronbach's α = 0.84<br>(Svanborg & Asberg 2001; Fantino, B., & Moore, N. 2009)
Hospital Anxiety and Depression Scale (HAD)	Validated in Swedish<br>test-retest reliability *r* = 0.72; Cronbachs α = 0.89-0.93<br>(Lisspers, J., et al. 1997)
Columbia Suicide Severity Rating Scale (C-SSRS)	Validated in Swedish<br>C-SSRS total score, AUC = 0.65 and a cut-off of 28.5 gave a sensitivity of 69% and a specificity of 54% in predicting a non-fatal or fatal suicide attempt<br>Cronbach's α = 0.73-0.95<br>(Posner et al., 2011; Lindh et al., 2018)
UKU side effect rating scale (UKU)	Validated<br>inter-rater reliability *r* = 0.07-0.80<br>(Lingjaerde et al., 1987; Lindström 2001)

Notes: Bergman, H., & Kallmen H. (2002). Alcohol use among Swedes and a psychometric evaluation of the alcohol use disorders identification test. Alcohol and Alcoholism, 37(3), 245–251. https://doi.org/10.1093/alcalc/37.3.245

Berman, A. H., Bergman, H., Palmstierna, T., & Schlyter, F. (2005). Evaluation of the Drug Use Disorders Identification Test (DUDIT) in Criminal Justice and Detoxification Settings and in a Swedish Population Sample. European Addiction Research, 11(1), 22-31. https://doi.org/10.1159/000081413

Bernstein DP, Fink L. Childhood Trauma Questionnaire. A retrospective self-report. Manual. The Psychological Corporation, Harcourt Brace & Company, San Antonio, TX 1998

Fantino, B., & Moore, N. (2009). The self-reported Montgomery-Asberg Depression Rating Scale is a useful evaluative tool in Major Depressive Disorder. BMC psychiatry, 9, 26. https://doi.org/10.1186/1471-244X-9-26

Gerdner, A., & Allgulander, C. (2009). Psychometric properties of the Swedish version of the Childhood Trauma Questionnaire-Short Form (CTQ-SF). Nordic journal of psychiatry, 63(2), 160–170. https://doi.org/10.1080/08039480802514366

For peer review only

Hildebrand M. (2015). The Psychometric Properties of the Drug Use Disorders Identification Test (DUDIT): A Review of Recent Research. Journal of substance abuse treatment, 53, 52–59. https://doi.org/10.1016/j.jsat.2015.01.008

Jokinen, J., Forslund, K., Ahnemark, E., Gustavsson, J. P., Nordström, P., & Asberg, M. (2010). Karolinska Interpersonal Violence Scale predicts suicide in suicide attempters. The Journal of clinical psychiatry, 71(8), 1025–1032. https://doi.org/10.4088/JCP.09m05944blu

Kalichman, S. C., & Rompa, D. (1995). Sexual sensation seeking and Sexual Compulsivity Scales: reliability, validity, and predicting HIV risk behavior. Journal of personality assessment, 65(3), 586–601. https://doi.org/10.1207/s15327752jpa6503_16

Kessler, R. C., Adler, L. A., Gruber, M. J., Sarawate, C. A., Spencer, T., & Van Brunt, D. L. (2007). Validity of the World Health Organization Adult ADHD Self-Report Scale (ASRS) Screener in a representative sample of health plan members. International journal of methods in psychiatric research, 16(2), 52–65. https://doi.org/10.1002/mpr.208

Lindh, Å.U., Waern, M., Beckman, K. et al. Short term risk of non-fatal and fatal suicidal behaviours: the predictive validity of the Columbia-Suicide Severity Rating Scale in a Swedish adult psychiatric population with a recent episode of self-harm. BMC Psychiatry 18, 319 (2018). https://doi.org/10.1186/s12888-018-1883-8

Lindström, E., Lewander, T., Malm, U., Malt, U. F., Lublin, H., & Ahlfors, U. G. (2001). Patient-rated versus clinician-rated side effects of drug treatment in schizophrenia. Clinical validation of a self-rating version of the UKU Side Effect Rating Scale (UKU-SERS-Pat). Nordic journal of psychiatry, 55 Suppl 44, 5–69. https://doi.org/10.1080/080394801317084428

Lingjaerde, O., Ahlfors, U. G., Bech, P., Dencker, S. J., & Elgen, K. (1987). The UKU side effect rating scale. A new comprehensive rating scale for psychotropic drugs and a cross-sectional study of side effects in neuroleptic-treated patients. Acta psychiatrica Scandinavica. Supplementum, 334, 1–100. https://doi.org/10.1111/j.1600-0447.1987.tb10566.x

Lisspers, J., Nygren, A., & Söderman, E. (1997). Hospital Anxiety and Depression Scale (HAD): some psychometric data for a Swedish sample. Acta psychiatrica Scandinavica, 96(4), 281–286. https://doi.org/10.1111/j.1600-0447.1997.tb10164.x

Långström N. Self-assessment of Sexual Interest (SSI). Unpublished questionnaire. 2010.

Molander, O., Wennberg, P., & Berman, A. H. (2021). The Gambling Disorders Identification Test (GDIT): Psychometric Evaluation of a New Comprehensive Measure for Gambling Disorder and Problem Gambling. Assessment, 10731911211046045. Advance online publication. https://doi.org/10.1177/10731911211046045

Posner, K., Brown, G. K., Stanley, B., Brent, D. A., Yershova, K. V., Oquendo, M. A., Currier, G. W., Melvin, G. A., Greenhill, L., Shen, S., & Mann, J. J. (2011). The Columbia-Suicide Severity Rating Scale: initial validity and internal consistency findings from three multisite studies with adolescents and adults. The American journal of psychiatry, 168(12), 1266–1277. https://doi.org/10.1176/appi.ajp.2011.10111704

Reid, R. C., Garos, S., & Carpenter, B. N. (2011). Reliability, Validity, and Psychometric Development of the Hypersexual Behavior Inventory in an Outpatient Sample of Men. Sexual Addiction & Compulsivity, 18(1), 30-51. https.//doi.org/10.1080/10720162.2011.555709

For peer review only

Rosen, R. C., Riley, A., Wagner, G., Osterloh, I. H., Kirkpatrick, J., & Mishra, A. (1997). The international index of erectile function (IIEF): a multidimensional scale for assessment of erectile dysfunction. Urology, 49(6), 822–830. https://doi.org/10.1016/s0090-4295(97)00238-0

Rosen, R. C., Cappelleri, J. C., & Gendrano, N., 3rd (2002). The International Index of Erectile Function (IIEF): a state-of-the-science review. International journal of impotence research, 14(4), 226–244. https://doi.org/10.1038/sj.ijir.3900857

Savard, J. (2021). Compulsive sexual behavior disorder: clinical characteristics and treatment with Naltrexone (PhD dissertation, Umeå universitetet). Retrieved from http://urn.kb.se/resolve?urn=urn:nbn:se:umu:diva-186811

Sheehan, D. V., Lecrubier, Y., Sheehan, K. H., Amorim, P., Janavs, J., Weiller, E., Hergueta, T., Baker, R., & Dunbar, G. C. (1998). The Mini-International Neuropsychiatric Interview (M.I.N.I.): the development and validation of a structured diagnostic psychiatric interview for DSM-IV and ICD-10. Journal of Clinical Psychiatry, 59 Suppl 20, 22-33;quiz 34-57

Stanford, M. S., Mathias, C. W., Dougherty, D. M., Lake, S. L., Anderson, N. E., & Patton, J. H. (2009). Fifty years of the Barratt Impulsiveness Scale: An update and review. Personality and Individual Differences, 47(5), 385–395. https://doi.org/10.1016/j.paid.2009.04.008

Svanborg P, Asberg M. A comparison between the Beck Depression Inventory (BDI) and the self-rating version of the Montgomery Asberg Depression Rating Scale (MADRS). J Affect Disord. 2001;64(2-3):203-16.

Öberg, K. G., Hallberg, J., Kaldo, V., Dhejne, C., & Arver, S. (2017). Hypersexual Disorder According to the Hypersexual Disorder Screening Inventory in Help-Seeking Swedish Men and Women With Self-Identified Hypersexual Behavior. Sexual medicine, 5(4), e229–e236. https://doi.org/10.1016/j.esxm.2017.08.001

For peer review only

SPIRIT
STANDARD PROTOCOL ITEMS: RECOMMENDATIONS FOR INTERVENTIONAL TRIALS

SPIRIT 2013 Checklist: Recommended items to address in a clinical trial protocol and related documents*

Section/item	Item No	Description	Addressed on page number
**Administrative information**			
Title	1	Descriptive title identifying the study design, population, interventions, and, if applicable, trial acronym	Title page
Trial registration	2a	Trial identifier and registry name. If not yet registered, name of intended registry	Abstract
	2b	All items from the World Health Organization Trial Registration Data Set	Link in abstract
Protocol version	3	Date and version identifier	4
Funding	4	Sources and types of financial, material, and other support	14
Roles and responsibilities	5a	Names, affiliations, and roles of protocol contributors	Title page
	5b	Name and contact information for the trial sponsor	Title page
	5c	Role of study sponsor and funders, if any, in study design; collection, management, analysis, and interpretation of data; writing of the report; and the decision to submit the report for publication, including whether they will have ultimate authority over any of these activities	14
	5d	Composition, roles, and responsibilities of the coordinating centre, steering committee, endpoint adjudication committee, data management team, and other individuals or groups overseeing the trial, if applicable (see Item 21a for data monitoring committee)	11

For peer review only

**Introduction**			
Background and rationale	6a	Description of research question and justification for undertaking the trial, including summary of relevant studies (published and unpublished) examining benefits and harms for each intervention	2-3
	6b	Explanation for choice of comparators	3
Objectives	7	Specific objectives or hypotheses	3-4
Trial design	8	Description of trial design including type of trial (eg, parallel group, crossover, factorial, single group), allocation ratio, and framework (eg, superiority, equivalence, noninferiority, exploratory)	4
**Methods: Participants, interventions, and outcomes**			
Study setting	9	Description of study settings (eg, community clinic, academic hospital) and list of countries where data will be collected. Reference to where list of study sites can be obtained	4
Eligibility criteria	10	Inclusion and exclusion criteria for participants. If applicable, eligibility criteria for study centres and individuals who will perform the interventions (eg, surgeons, psychotherapists)	5
Interventions	11a	Interventions for each group with sufficient detail to allow replication, including how and when they will be administered	6-8 + figure 1
	11b	Criteria for discontinuing or modifying allocated interventions for a given trial participant (eg, drug dose change in response to harms, participant request, or improving/worsening disease)	11
	11c	Strategies to improve adherence to intervention protocols, and any procedures for monitoring adherence (eg, drug tablet return, laboratory tests)	9
	11d	Relevant concomitant care and interventions that are permitted or prohibited during the trial	11
Outcomes	12	Primary, secondary, and other outcomes, including the specific measurement variable (eg, systolic blood pressure), analysis metric (eg, change from baseline, final value, time to event), method of aggregation (eg, median, proportion), and time point for each outcome. Explanation of the clinical relevance of chosen efficacy and harm outcomes is strongly recommended	9 + Table 3
Participant timeline	13	Time schedule of enrolment, interventions (including any run-ins and washouts), assessments, and visits for participants. A schematic diagram is highly recommended (see Figure)	Figure 1

For peer review only - http://bmjopen.bmj.com/site/about/guidelines.xhtml

Sample size	14	Estimated number of participants needed to achieve study objectives and how it was determined, including clinical and statistical assumptions supporting any sample size calculations	11
Recruitment	15	Strategies for achieving adequate participant enrolment to reach target sample size	6, 11

Methods: Assignment of interventions (for controlled trials)

Allocation:

Sequence generation	16a	Method of generating the allocation sequence (eg, computer-generated random numbers), and list of any factors for stratification. To reduce predictability of a random sequence, details of any planned restriction (eg, blocking) should be provided in a separate document that is unavailable to those who enrol participants or assign interventions	8
Allocation concealment mechanism	16b	Mechanism of implementing the allocation sequence (eg, central telephone; sequentially numbered, opaque, sealed envelopes), describing any steps to conceal the sequence until interventions are assigned	8
Implementation	16c	Who will generate the allocation sequence, who will enrol participants, and who will assign participants to interventions	8
Blinding (masking)	17a	Who will be blinded after assignment to interventions (eg, trial participants, care providers, outcome assessors, data analysts), and how	N/A
	17b	If blinded, circumstances under which unblinding is permissible, and procedure for revealing a participant's allocated intervention during the trial	N/A

Methods: Data collection, management, and analysis

Data collection methods	18a	Plans for assessment and collection of outcome, baseline, and other trial data, including any related processes to promote data quality (eg, duplicate measurements, training of assessors) and a description of study instruments (eg, questionnaires, laboratory tests) along with their reliability and validity, if known. Reference to where data collection forms can be found, if not in the protocol	6, 9, Table 3, supplement table 1
	18b	Plans to promote participant retention and complete follow-up, including list of any outcome data to be collected for participants who discontinue or deviate from intervention protocols	11

Data management	19	Plans for data entry, coding, security, and storage, including any related processes to promote data quality (eg, double data entry; range checks for data values). Reference to where details of data management procedures can be found, if not in the protocol	12
Statistical methods	20a	Statistical methods for analysing primary and secondary outcomes. Reference to where other details of the statistical analysis plan can be found, if not in the protocol	11
	20b	Methods for any additional analyses (eg, subgroup and adjusted analyses)	11
	20c	Definition of analysis population relating to protocol non-adherence (eg, as randomised analysis), and any statistical methods to handle missing data (eg, multiple imputation)	11
**Methods: Monitoring**			
Data monitoring	21a	Composition of data monitoring committee (DMC); summary of its role and reporting structure; statement of whether it is independent from the sponsor and competing interests; and reference to where further details about its charter can be found, if not in the protocol. Alternatively, an explanation of why a DMC is not needed	11
	21b	Description of any interim analyses and stopping guidelines, including who will have access to these interim results and make the final decision to terminate the trial	11
Harms	22	Plans for collecting, assessing, reporting, and managing solicited and spontaneously reported adverse events and other unintended effects of trial interventions or trial conduct	8, 9, 11, 12
Auditing	23	Frequency and procedures for auditing trial conduct, if any, and whether the process will be independent from investigators and the sponsor	11
**Ethics and dissemination**			
Research ethics approval	24	Plans for seeking research ethics committee/institutional review board (REC/IRB) approval	12
Protocol amendments	25	Plans for communicating important protocol modifications (eg, changes to eligibility criteria, outcomes, analyses) to relevant parties (eg, investigators, REC/IRBs, trial participants, trial registries, journals, regulators)	4, 12

For peer review only - http://bmjopen.bmj.com/site/about/guidelines.xhtml

Consent or assent	26a	Who will obtain informed consent or assent from potential trial participants or authorised surrogates, and how (see Item 32)	11
	26b	Additional consent provisions for collection and use of participant data and biological specimens in ancillary studies, if applicable	Supplement material (Patient consent form)
Confidentiality	27	How personal information about potential and enrolled participants will be collected, shared, and maintained in order to protect confidentiality before, during, and after the trial	12, 14
Declaration of interests	28	Financial and other competing interests for principal investigators for the overall trial and each study site	14
Access to data	29	Statement of who will have access to the final trial dataset, and disclosure of contractual agreements that limit such access for investigators	14
Ancillary and post-trial care	30	Provisions, if any, for ancillary and post-trial care, and for compensation to those who suffer harm from trial participation	11-12
Dissemination policy	31a	Plans for investigators and sponsor to communicate trial results to participants, healthcare professionals, the public, and other relevant groups (eg, via publication, reporting in results databases, or other data sharing arrangements), including any publication restrictions	12
	31b	Authorship eligibility guidelines and any intended use of professional writers	12
	31c	Plans, if any, for granting public access to the full protocol, participant-level dataset, and statistical code	14
**Appendices**			
Informed consent materials	32	Model consent form and other related documentation given to participants and authorised surrogates	Supplement material (patient consent form)
Biological specimens	33	Plans for collection, laboratory evaluation, and storage of biological specimens for genetic or molecular analysis in the current trial and for future use in ancillary studies, if applicable	Supplement material (patient consent form)

For peer review only - http://bmjopen.bmj.com/site/about/guidelines.xhtml

*It is strongly recommended that this checklist be read in conjunction with the SPIRIT 2013 Explanation & Elaboration for important clarification on the items. Amendments to the protocol should be tracked and dated. The SPIRIT checklist is copyrighted by the SPIRIT Group under the Creative Commons "Attribution-NonCommercial-NoDerivs 3.0 Unported" license.

For peer review only
